# Impact of nanofillers on vitrimerization and recycling strategies: a review

**DOI:** 10.1039/d5na00183h

**Published:** 2025-07-23

**Authors:** Sourav Ghosh, Amrita Chatterjee, Nilanjan Dey, Sunidhi Mishra, Shakshi Bhardwaj, Shiva Singh, Ujjal Tewary, Satyam Sahay, Madhuchhanda Maiti, Pradip K. Maji

**Affiliations:** a Department of Polymer and Process Engineering, Indian Institute of Technology Roorkee Saharanpur Campus Saharanpur-247001 India pradip@pe.iitr.ac.in +91-7895965010; b Enterprise Technology and Engineering Center, John Deere India Pvt Ltd Tower-XIV, Cyber City, Hadapsar Pune Maharashtra-411013 India

## Abstract

The growing complexity of the waste from polymer nanocomposite materials has brought attention to the pressing need for creative recycling techniques. Vitrimerization, based on dynamic covalent bond exchange mechanisms, offers a transformative method to improve the recyclability, reprocessability, and long-term durability of polymer-based nanocomposites. This paper conveys a complete overview of vitrimer chemistry, vitrimer processing techniques, and their integration with diverse nanofillers. The significance of nanofiller addition in modifying vitrimer network dynamics, mechanical performance, thermal stability, and self-healing capacities is critically investigated. Case studies and performance tests illustrate the major advantages of vitrimerized nanocomposites, including better mechanical characteristics, energy-efficient recyclability, and prolonged service life. Furthermore, the study investigates the industrial significance of materials based on vitrimers in fields including biomedical engineering and the aerospace and automotive sectors. The potential of vitrimerization to achieve sustainable, circular material lifecycles is highlighted by a comparison with conventional recycling techniques. Finally, future research topics and obstacles linked to large-scale deployment are discussed. This review seeks to serve as a foundational reference for researchers exploring vitrimer-based recycling technologies for high-performance as well as ecologically responsible polymer nanocomposites.

## Abstract 2

Innovative recycling of polymer nanocomposites enabled by vitrimerization, highlighting the role of nanofillers in enhancing network dynamics, recyclability, mechanical strength, and sustainable material lifecycles.

## Graphical abstract

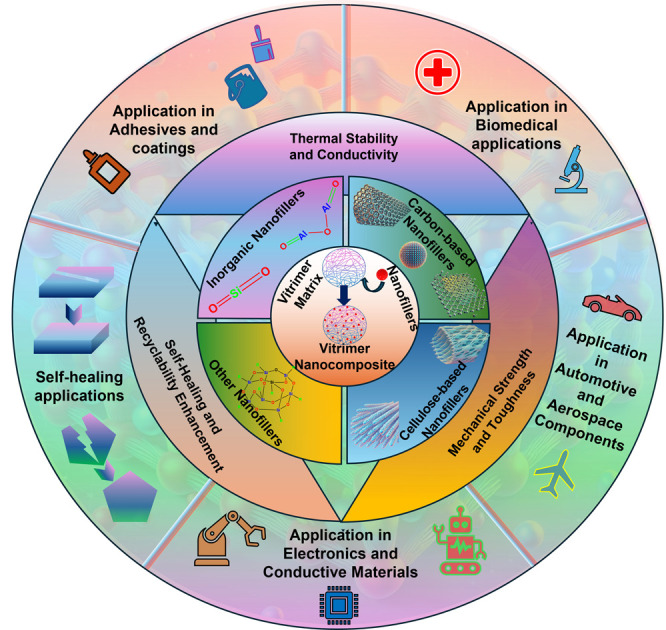

## 1. Introduction

In recent decades, traditional cross-linked thermosetting polymeric materials have gained a lot of attention due to their enhanced thermal stability, mechanical properties, and chemical resistance. These materials are crucial in many fields and industries due to their exceptional properties.^1–3^ Despite their superior mechanical strength, chemical resistance, and thermal stability, traditional thermosetting polymers without nanofillers are naturally brittle, cannot be recycled, and have limited multifunctional uses. However, once thermally processed into their final shape, these thermosetting polymer matrices undergo irreversible cross-linking.^4,5^ Because of this, most materials are naturally insoluble and infusible, and there is substantial concern regarding the recyclability of traditional polymer nanocomposites.^6,7^ Even though these features are supposed to be substantial benefits for thermoset matrix composites throughout their service life, the difficulty in recycling them renders them considerable drawbacks at the end of their useful life. Nanocomposites with improved mechanical, thermal, electrical, and barrier characteristics over their bulk counterparts are formed when nanoscale fillers are introduced into the polymer matrix.^8–10^ Thermoset polymers are frequently reinforced with carbon nanotubes, graphene, and various other nanofillers to increase their mechanical, thermal, and electrical characteristics.^11,12^ This makes thermoset composites more complex to recycle, rework, or reprocess. Separating the reinforcement from the matrix is one of the difficulties in the recycling process.^13,14^ Thermolysis, solvolysis, or other procedures are utilized in modern-day operations to recover reinforcement.^15–18^ However, the mechanical properties are considerably modified. As a result, these fillers cannot be recycled again and used for the same applications for which they were initially designed.^19–21^ This constraint has been solved by the construction of a new class of materials called “vitrimers”.^22,23^ The formation of vitrimers requires the presence of dynamic covalent bonds capable of generating covalent adaptable networks (CAN) that stimulate exchange reactions. As a result, vitrimer systems can undergo reversible reactions, allowing bonds to form and break in response to external stimuli while maintaining the overall number of chemical linkages. This distinguishing feature is the result of the investigation and analysis of a number of dynamic covalent bonds in this system, including esters,^24^ carbonates,^25^ carbamates,^26^ imines,^27^ boron esters,^28^ diboroxine,^29^ disulfides,^30^ and others.^31^

Vitrimers' outstanding stability and processability emphasize their versatility, which has led to applications in recyclable polymers and reprocessable polymers that promote sustainable practices.^32–35^ Furthermore, vitrimers are easily processable for alterations and repairs in coatings, adhesives, and reshapable polymers.^36–39^ Because of their recyclability, which is lacking in most nanocomposite materials, vitrimers play an essential role in nanocomposites. They have a broad influence in a variety of applications. Vitrimers can thus be used as alternatives to the sustainable polymers that are now used in nanocomposite products.^40–43^ Vitrimers have had a significant impact on the development of scientific applications due to their adaptability in a range of fields. To promote the continual growth of materials science, ongoing research activities are focused on identifying new applications and improving the functioning of vitrimer-based materials. In terms of sustainability, self-healing, and recyclability, vitrimer nanocomposites surpass typical polymer nanocomposites and provide noticeable advantages.^41^ Despite their many advantages, traditional polymer nanocomposites often feature irreversible cross-linking that makes recycling or repair problematic. This leads to the generation of waste and serious environmental consequences. On the other hand, dynamic covalent bonds that, in certain settings, encourage bond exchange processes are exploited by vitrimer nanocomposites, allowing for greater recyclability, self-healing, and reprocessing. The structural integrity of traditional composites and the flexibility and durability of vitrimers are combined in these materials, making them suitable for applications that demand longevity, functionality, and environmental responsibility. Furthermore, it is feasible to adjust the dynamic network of vitrimers, opening up new possibilities in domains like electronics, healthcare, and aerospace, where standard composites are unsuited. Although several excellent reviews have studied vitrimer chemistry and applications, relatively few have focused on how nanofillers influence vitrimerization behavior and the recyclability of vitrimer nanocomposites.^41,44–46^ This junction is significant, since nanofillers not only reinforce vitrimer networks but can also modify exchange kinetics, mechanical characteristics, and the effectiveness of recycling and self-healing.^47–50^ To contextualize the present study within existing literature, a comparison of vitrimer and vitrimer nanocomposite-related review papers is presented in Table 1. Furthermore, understanding the compatibility of various nanofillers with vitrimer matrices is vital for building next-generation sustainable composites. This study intends to overcome this essential gap by giving a complete assessment of the influence of nanofillers on vitrimerization and recycling techniques. By concentrating on this underexplored synergy between nanofillers and vitrimer networks, our study gives unique insights into the design of recyclable, high-performance nanocomposites that are consistent with global sustainability goals.

**Table 1:** Comparative analysis of key vitrimer and vitrimer nanocomposite review papers highlighting the focus, findings, and relevance to the present stud*y*

S. no.	Title	Main focus	Key contributions	Ref.
1	Vitrimer composites: status and future challenges	Recycling thermoset composites *via* vitrimerization	Provides an overview of vitrimer composites and recycling strategies, including early industrial efforts	41
2	From landfilling to vitrimer chemistry in the rubber life cycle	Vitrimerization for rubber recycling	Discusses dynamic networks in rubbers, including Diels–Alder and vitrimer chemistries	51
3	Understanding vitrimer properties through various aspects of inhomogeneity	Network inhomogeneity in vitrimers	Explores how inhomogeneous structures impact vitrimer behavior	52
4	Self-healable fiber-reinforced vitrimer composites: overview and future prospects	Fiber-reinforced vitrimer composites	Reviews recyclability, healing, and structural reinforcement	53
5	Carbon material/vitrimer composites: towards sustainable, functional, and high-performance crosslinked polymeric materials	Carbon-based vitrimers (CNT, graphene, *etc.*)	Highlights enhanced electrical, thermal, and mechanical properties	54
6	Vitrimer nanocomposites for highly thermal conducting materials with sustainability	Thermal conductivity in vitrimer composites	Focuses on thermally conductive nanocomposites	55
7	On the welding of vitrimers: chemistry, mechanics and applications	Welding of vitrimer materials	Discusses vitrimer welding chemistry, testing, and design strategies	56
8	Vitrimers: permanent organic networks with glass-like fluidity	Chemistry and behavior of vitrimers	Provides a mini-review on chemical mechanisms, network behavior, and processing	57
9	Vitrimers: associative dynamic covalent adaptive networks in thermoset polymers	ADCAN networks in thermosets	Highlights associative bond mechanisms and self-healing in thermosets	58
10	Next-generation vitrimer composites for future mobility: balancing sustainability and functionality – a perspective	Mobility-focused vitrimer composites	Reviews fillers, matrices, and vitrimers for automobile/aerospace applications	59
11	Functional epoxy vitrimers and composites	Epoxy-based vitrimer systems	Details epoxy vitrimer chemistry, network mechanics, and applications	22
12	Impact of nanofillers on vitrimerization and recycling strategies: a review	Comprehensive review of vitrimer chemistry, nanofillers, performance, and applications	Discusses associative chemistry, nanofiller integration, mechanical/thermal tests, industrial relevance, and future challenges	Present review

This paper intends to give a full introduction to vitrimerization and its implications for the recycling of polymer-based nanocomposites. The topic begins with the fundamental ideas of vitrimer chemistry, including the dynamics of covalent networks and bond exchange processes. The accompanying sections give a full evaluation of the compatibility of vitrimerization with various nanofillers and their role in boosting the performance of recycled composites. The efficacy and benefits of vitrimer-based recycling are illustrated through case studies and practical examples. The research examines the economic and environmental advantages of vitrimerization, emphasizing its potential alignment with global sustainability goals. It emphasizes the distinct benefits of vitrimerization regarding energy efficiency, material longevity, and reduced carbon footprint by comparing it to conventional recycling techniques. The final half of the discussion offers insights into emerging research avenues and the challenges associated with broadening the industrial use of vitrimer-based recycling, highlighting future prospects and improvements in vitrimer technology. In conclusion, vitrimerization, an emerging domain in materials science, has the potential to revolutionize the recycling and lifecycle management of polymer nanocomposites. By extensively studying its tenets, applications, and advantages, this paper intends to add to the burgeoning body of research on sustainable materials and encourage new discoveries in this revolutionary domain.

## 2. Fundamentals of vitrimer-based nanocomposites

The increased demand for sophisticated materials in modern industries has led to the widespread usage of nanocomposites,^60^ which combine polymers with nanofillers to produce superior mechanical, thermal, and functional qualities in the polymer.^61^ Despite their performance advantages, conventional nanocomposites, particularly those based on thermoset polymers, present severe sustainability and recycling issues.^62–64^ These materials are often distinguished by permanent crosslinked networks that render them non-reprocessable, non-recyclable, and very resistant to degradation, resulting in waste accumulation and major environmental concerns. Conventional recycling procedures, such as mechanical reprocessing or chemical depolymerization, are frequently ineffectual for these materials, owing to the difficulties of separating nanofillers from the polymer matrix and the irreversible nature of the crosslinked structure.^65–67^ As enterprises work to move to a circular economy model in which resources are reused, waste is reduced, and sustainability is prioritized, the limitations of traditional nanocomposites offer a significant barrier to attaining these objectives. A revolutionary solution to these recycling challenges for traditional nanocomposites is offered by vitrimerization, which is a unique technique for adding dynamic covalent connections into polymer networks.^45,68–70^ Under specific conditions, such as heat or the presence of catalysts, a vitrimer is a type of polymer that may undergo bond exchange processes, enabling reshaping, reprocessing, and even self-healing without compromising mechanical integrity. These materials provide a special combination of enhanced functionality and recyclability when combined with nanofillers to create vitrimer-based nanocomposites.^71,72^ Vitrimer-based nanocomposites are superior for applications needing durability and sustainability because, in contrast to conventional thermosets, they preserve the strength and stability of crosslinked networks while permitting several reprocessing cycles. This new paradigm addresses a number of crucial concerns in the materials lifecycle. Vitrimerization eliminates waste associated with end-of-life products by enabling closed-loop recycling, which allows materials to be entirely recovered and reused.^73^ Second, the dynamic nature of vitrimer linkages allows for the separation of nanofillers from the polymer matrix, increasing the recyclability of both components. Finally, the versatility of vitrimer chemistry allows for the production of materials customized to specific performance and environmental criteria, which aligns with the ideals of a circular economy.

### 2.1 Nanofillers for vitrimer matrix property enhancement

The sophisticated, versatile, recyclable, and adaptable materials known as vitrimer-based nanocomposites are made by combining dynamic vitrimer matrices with nanofillers. The special characteristics of vitrimer matrices, which are polymers with dynamic covalent bonds that permit bond exchange under certain conditions, include self-healing, reprocessability, and adaptability to industrial and environmental demands.^48,74,75^ There are a number of vitrimer matrices that are frequently utilized, and each one has its own set of benefits. For demanding applications in aerospace, automotive, and industrial components, epoxy-based vitrimers are highly regarded for their great mechanical strength and outstanding thermal stability. An eco-friendly option for uses like packaging and throwaway items is vitrimers made of polyester, which are both flexible and biodegradable.^76,77^ Coatings, adhesives, and other flexible goods that demand resilience can benefit from the usage of polyurethane vitrimers due to their elasticity and impact resistance.^78–80^ Polyamide vitrimers, on the other hand, are ideal for chemical storage systems and heavy-duty industrial components because of their exceptional toughness, resistance to chemicals, and longevity.^81,82^ In addition to vitrimer matrices, nanofillers play a crucial role in improving the overall performance of nanocomposites. They provide qualities including strength and conductivity. Carbon-based nanofillers, such as carbon nanotubes (CNTs), graphene, and carbon black, are widely used for their exceptional ability to improve electrical and thermal conductivity as well as mechanical reinforcement, making them valuable in electronics, conductive coatings, and lightweight structural applications.^83–85^ Metal oxide nanofillers, including titanium dioxide, aluminum oxide, and zinc oxide, are typically included to enhance thermal stability, UV resistance, and flame retardancy, making them appropriate for packaging, construction, and high-performance coating applications.^86–88^ Nanofillers made of clay, such as halloysite and montmorillonite, are highly prized in the packaging, painting, and protective coating industries for their capacity to increase fire resistance, dimensional stability, and barrier qualities. Organic nanofillers, such as cellulose nanocrystals and lignin, are gaining popularity due to their sustainability, biodegradability, and ability to deliver bio-based increases in strength and thermal properties, matching the growing need for ecologically benign materials.^89–91^ These vitrimer matrices and nanofillers can be mixed in varied ways to develop materials customized for specific applications, overcoming many of the recycling issues experienced by standard nanocomposites. Because of their permanently crosslinked structure, reprocessing and reshaping are hindered. Conventional nanocomposites, usually composed of thermoset polymers, are notoriously difficult to recycle. In contrast, vitrimerization allows the incorporation of dynamic covalent chemistry, providing reprocessable materials that can be molded, mended, or recycled several times without losing performance.^92,93^ This versatility not only makes vitrimer-based nanocomposites environmentally friendly but also decreases the requirement for single-use resources, supporting circular economy aims. The potential uses of these materials are vast and include lightweight, high-strength components in the automotive and aerospace sectors, flexible and recyclable conductive sheets for electronics, self-healing medical implants, and biocompatible scaffolds for tissue engineering. In the energy sector, these materials find employment in battery separators, fuel cells, and other energy storage devices, where excellent performance and recyclability are crucial. The compatibility of vitrimer matrices with a wide range of nanofillers also enables the modification of features, such as improved toughness, thermal stability, and barrier resistance, to satisfy the specific needs of various industries. Overall, the combination of dynamic vitrimer chemistry with sophisticated nanofillers represents a breakthrough approach to generating high-performance, sustainable materials that alleviate both technical and environmental concerns in a wide range of applications.

## 3. Chemistry involved in vitrimer nanocomposites and their recycling: the science behind the technique

Materials science has emerged at the forefront to provide creative answers to pressing global environmental issues. The emergence of vitrimers, a novel family of polymers that combine the recyclability of thermoplastics with the stiffness of thermosets, is one of the most exciting advances. Especially in the field of nanocomposites, these novel materials have the ability to totally change the conventional methods of production, application, and reprocessing of polymers.^94^ The basis of vitrimers is dynamic covalent chemistry, which produces materials with unique mechanical and thermal characteristics by reversible bond exchange methods. The complex chemistry of vitrimers, recyclable processes, and the effect of their incorporation into nanocomposites are examined in this paper. Moreover, it covers the real-life implications and the circular economy.^95–97^ This thorough analysis has focused on highlighting the revolutionary potential of vitrimers. This review shows where these vitrimers differ from conventional polymers and the benefits they provide for producing high-performance, sustainable materials.

### 3.1 Dynamic covalent bonds: the backbone of vitrimers

**Fig. 1:**
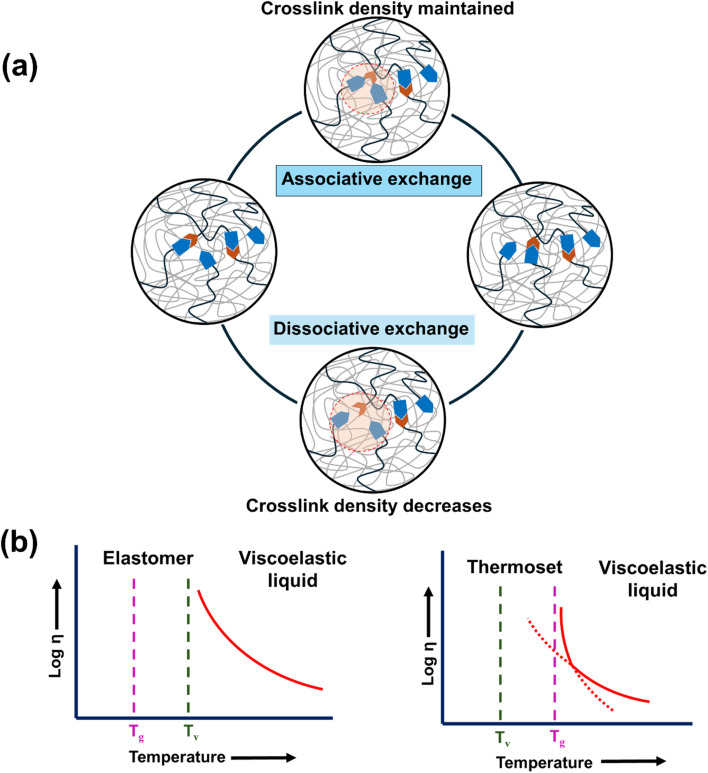
(a) Associative and dissociative bond exchange reaction in a vitrimer matrix, (b) idealised viscosity *vs.* temperature curve for vitrimer matrix when *T*_g_ < *T*_v_ and *T*_g_ > *T*_v_.

Dynamic covalent bonds have transformed polymer chemistry by introducing reversible covalent interactions into polymeric materials to meet the global demand for sustainable materials. In honor of Lehn's groundbreaking research on dynamers, the concept of “dynamic covalent chemistry” was introduced.^98^ These dynamic interactions give the materials an impressive array of properties, such as self-healing, recyclability, stress relaxation, and adaptability. By combining the mechanical and thermal strength of thermosets with the recyclability and reprocessability of thermoplastics, vitrimers, a special subclass of covalent adaptable networks (CANs), bridge the gap between these two material classes.^99^ Vitrimers are distinguished by their ability to retain network integrity while carrying out dynamic bond exchange reactions in the presence of catalysts and thermal conditions *via* two key mechanisms: dissociative and associative bond exchange (Fig. 1a). In the associative mechanism, the bond exchange process generates a new covalent link before cleaving the old one, sustaining network linkages and avoiding structural breakdown. Transesterification reactions are an example of this process.^100,101^ In most cases, with the aid of Lewis acids or bases, alcohol groups nucleophilically attack ester bonds in these processes. The first alcohol group is released, causing the tetrahedral intermediate to disintegrate. As an additional illustration, consider the dynamic imine bond exchange when nucleophilic amines interact with carbonyl groups. This approach yields reversible imine synthesis, which is extremely beneficial for self-healing materials due to its efficient exchange dynamics.^102^ For applications that call for strong and durable materials, the associative mechanism offers outstanding network stability during reprocessing, minimal risk of depolymerization, and high mechanical characteristics.^103^ To achieve the best performance, however, precise temperature and catalyst concentration control are necessary, and it is often linked to slower reaction kinetics and higher activation energy requirements. In the dissociative process, an existing covalent bond is broken at the start of the exchange reaction, which causes a temporary state in which network connectivity is disrupted until a new bond is established to restore the structure. This method's quicker response kinetics and lower energy barriers make it helpful for applications requiring instantaneous stress relaxation or flow, but it can also result in a temporary loss of mechanical integrity and a higher risk of depolymerization at high temperatures.^104,105^ Dynamic disulfide bonds, which can be broken and reformed by thermal activation or redox reactions, and reversible Diels–Alder reactions, which enable bonds to be broken and reformed by thermally reversible cycloaddition processes, are examples of dissociative mechanisms.^106^ The original covalent bond is restored, for instance, when the diene and dienophile components split at high temperatures and reconnect when cooled. Due to the interplay of these mechanisms, vitrimers have special qualities such as thermal stability, flexibility, self-healing, and recyclability, making them adaptable materials for various uses. Associative processes in self-healing materials provide strong and reliable restoration, while dissociative mechanisms offer quicker reaction times for less critical applications. Dynamic covalent bonds in vitrimers are key to their recyclability and adaptability, with their behavior strongly dependent on transition temperatures.

The physical characteristics of thermally activated vitrimers vary with temperature. Two important transition temperatures are used to characterize distinct behaviors. First, the material becomes mouldable at the glass transition temperature (*T*_g_), where segmental movement of the polymer chain takes place. The second is the topological freezing transition temperature (*T*_v_), at which the material changes from a solid to a liquid due to rapid bond exchange and polymer flow. This temperature is typically chosen to represent the point at which a viscosity of 10^12^ Pa s is achieved. *T*_g_ and *T*_v_ have independent temperatures because of their distinct interactions. The vitrimer changes from a stiff solid below *T*_g_ to an elastic solid between *T*_g_ and *T*_v_ to a viscoelastic liquid above *T*_v_. Usually, *T*_g_ is lower than *T*_v_. The Arrhenius equation can be used to depict viscosity reduction since chemical exchanges control flow at temperatures higher than *T*_v_. In certain situations, especially for thermoset materials, *T*_v_ may be lower than *T*_g_ (Fig. 1b).^107,108^ Under such conditions, no significant segmental motion takes place below *T*_g,_ resulting in no exchange reactions. The network is, therefore, fixed. Vitrimers can be made for various applications by changing *T*_g_ and *T*_v_ with different catalysts or polymer mixes. These materials are useful in high-temperature aerospace settings and for recycling with less energy. Catalysts like enzymes and organometallic complexes help to improve how the bonds work by reducing the temperatures needed for reactions and making them more selective. This careful tuning not only broadens the uses of vitrimers but also opens up new possibilities in material development, allowing for self-healing products, adjustable adhesives, and eco-friendly polymer production, among others.

### 3.2 Mechanisms involved in vitrimer nanocomposite recycling

Vitrimer recycling provides circular and closed-loop recycling pathways due to dynamic covalent bonds, which can modify the polymer network so that, at the time of recycling, we don't need to damage the network.^109,110^ As a result, the substance can be fixed, created, or utilized repeatedly without requiring new monomers or additives. Recycling vitrimers is less energy-intensive and better for the environment than recycling thermoplastics chemically. Reprocessing preserves the polymer matrix and nanofillers, benefiting both the circular economy and green chemistry. Also, thermally repairing microcracks or damage can extend the life of vitrimer nanocomposites while reducing the frequency of replacements. Vitrimers can be cut or ground into tiny pieces, reshaped, and reformed into new specimens while maintaining their mechanical and physicochemical characteristics, including glass transition temperature (*T*_g_), elastic modulus, thermal stability, tensile strength, and gel content, thanks to their flowability under heat or other stimuli.^111^ However, regular recyclability assessments often overlook side reactions that convert dynamic cross-links into static ones without altering the overall cross-link density, even though these reactions can have an impact on long-term performance. Selective breakage of dynamic bonds can address this issue and provide precise molecular-level information on characteristics such as molar mass and distribution, resulting in accurate recyclability evaluations.^112–114^ In vitrimer composites, such as polyimine, polyester, and vinylogous urea-based systems, this method has also made it possible to recycle them in a closed-loop manner, recovering and reusing fibers (such as carbon or glass) and polymer matrices to create new materials with the same qualities as the original material.^115–117^ The compatibility of vitrimer nanocomposite recycling with solvent-free, thermomechanical processing methods is another noteworthy feature.^118^ Industrially scaled techniques like compression molding, extrusion, and injection molding can be used to reprocess vitrimer nanocomposites, in contrast to conventional thermoset recycling processes like pyrolysis or solvolysis, which frequently result in fiber deterioration or loss of material functionality. These processes enable the reshaping or reformation of old materials into new forms by activating the bond exchange with heat and moderate pressure. It is possible to adjust the reprocessing conditions to maintain structural integrity and functionality, enabling repeated recycling with no degradation in mechanical performance.^119^ Vitrimer nanocomposites have demonstrated their durability and reusability in a number of documented scenarios by undergoing multiple recycling cycles with over 50% retention in tensile strength and flexural modulus.^83,84,120^ Vitrimers provide flexible, environmentally friendly solutions for sectors including consumer products, automotive, and aerospace through advanced characterization techniques and ideal processing conditions. By minimizing waste and preserving resources, vitrimers support the circular economy.

### 3.3 Advantages of chemistry in vitrimer matrix preparation

The chemistry underlying vitrimer matrix preparation provides several significant benefits that set vitrimer matrix preparation apart from traditional thermoset and thermoplastic systems. The addition of an associative dynamic covalent connection to a network of crosslinked polymers is the fundamental process of vitrimer technology.^23,121^ These bonds, which include vinylogous urethane couplings, disulfide exchange, transesterification, and imine exchange, allow for reversible network rearrangement while preserving the material's structural integrity. Without sacrificing the rigidity and chemical resistance characteristic of thermosets, this dynamic behavior gives the vitrimer shape memory, self-healing, and thermal reprocessability.^103,122^ Bond exchange kinetics, processing temperatures, and mechanical qualities can all be precisely adjusted by selecting the right monomers, functional groups, and catalysts. Furthermore, vitrimer chemistry is highly modular and versatile, allowing for the creation of networks using epoxies, polyesters, urethanes, and other materials.^123,124^

**Fig. 2:**
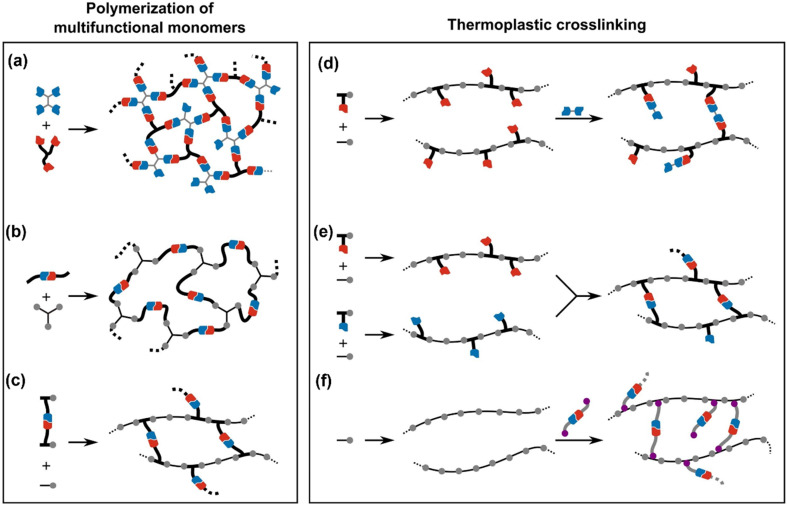
Synthetic strategies for the fabrication of the vitrimer matrix *via* polymerization of the multifunctional group and thermoplastic crosslinking. (a) Step-growth process of the vitrimer matrix. (b) Already contain the dynamic covalent linkages for bond exchange. (c) By using a bifunctional crosslinker with a dynamic covalent bond. (d and e) Solution and melt mixing of multifunctional molecule or a polymer. (f) Dynamic bond introduced *via* post-polymerization. Reproduced with permission from ref. 23 Copyright 2020 Elsevier B.V.

Polymerization of multifunctional monomers and thermoplastic cross-linking are the two primary methods for vitrimer matrix preparation. The first tactic is to cure a combination of monomers with several functions in order to create a network with dynamic covalent links. In the step-growth process of the vitrimer matrix, the newly created network^125–129^ (Fig. 2a) or at least one monomer^130–132^ may already contain the dynamic covalent linkages for bond exchange reactions (Fig. 2b). Another scenario is to change the composition of resins or add a catalyst that promotes exchange reactions, which can transform some commercial resins into vitrimers.^127,133^ Nicolaÿ *et al.* showed that the simplest way to conduct chain-growth polymerizations is by using a bifunctional crosslinker with a dynamic covalent bond to conduct a copolymerization for vinyl monomers (Fig. 2c).^134^ In order to synthesize a vitrimer matrix from multifunctional monomers, the active species participating in the polymerization process and the various monomers used in the polymerization must be compatible with each other and with dynamic covalent chemistry. In this technique, the vitrimer matrix is generated in a single step, which is beneficial from a reaction point of view. One of the potential issues is the requirement to remove the unreacted monomers or residual solvents from the vitrimer matrix.

The second strategy is to convert thermoplastic polymers into vitrimers. Two main scenarios exist. First, the dynamic functions that are engaged in the exchange process are internally present in the backbone of the polymers or as pendant functions. As for the transesterification exchange reaction, ester and alcohol groups are present internally in the epoxy and polyester-based vitrimer matrix.^135–137^ Similarly, the alkene bond of polydiene-based vitrimers is present internally for alkene metathesis.^138^ Otherwise, dynamic functionalities can be inserted on purpose during the production of the polymers by copolymerizing a comonomer containing the exchangeable group.^112,139–143^ After that, the functional thermoplastic polymers are cross-linked into vitrimers, either in solution or in melt, using a multifunctional molecule or a polymer (Fig. 2d and e). This technique gives the maximum versatility regarding vitrimer matrices, dynamic bonds, and synthesis conditions. Existing thermoplastics can also be transformed into vitrimers even if they do not carry any functional groups capable of dynamic exchange reactions. In such a scenario, the dynamic links are introduced *via* post-polymerization functionalization (Fig. 2f).^112,113^ The chemistry required to attach the crosslinkers depends on the reactivity of the parent polymers and should also be compatible with the exchangeable links. This approach is particularly interesting since it directly converts conventional polymers into vitrimers without changing existing and efficient syntheses. However, this approach can be very challenging due to the low reactivity of the polymers to be transformed (*e.g.*, polyolefins and fluoropolymers) or because of their low solubility and high melting temperature [*e.g.*, poly(ethylene terephthalate) and polyamides], which significantly limit the processing parameter window. In such instances, reactive extrusion is a useful strategy to accomplish this functionalization, which generally acts *via* radical-initiated reactions.

### 3.4 Bond exchange mechanisms: enabling recyclability

Vitrimers are dynamic polymer networks that employ exchangeable covalent connections to provide self-healing, reprocessability, and stress relaxation while maintaining structural integrity in a variety of environments. These distinguishing characteristics are the result of dynamic covalent processes, which enable bond breakage and reformation without altering the network's overall makeup. Many degenerate methods, including transesterification, imine exchange, disulfide exchange, boronic ester exchange, urethane exchange, and Diels–Alder reactions, have been effectively used in vitrimer synthesis.^144–148^ Depending on the application and processing requirements, each form of bond exchange has unique benefits. Dynamic covalent bonds may also be incorporated into thermoset polymers using modifications to traditional step-growth polymerization techniques such as ester, carbonate, or urethane synthesis.^149,150^ This method streamlines vitrimer manufacture by introducing components or modifications during synthesis that allow for dynamic behavior under certain conditions. Alternatively, these bonds may be included in thermoplastic cross-linking systems, although extra measures may be required to prevent interference between dynamic bond production and the main cross-linking process.

Catalysts are important in changing the performance of vitrimers because they control the lifespan and kinetics of dynamic bonds. The activation energy needed for bond exchange is affected by both the structure and concentration of catalysts, altering the thermal, mechanical, and chemical characteristics of vitrimers.^151^ Balancing these issues with material performance is critical for developing workable vitrimer formulations, particularly for applications requiring long-term durability or environmental stability. Static cross-links may be used deliberately by vitrimer designers to improve mechanical properties without sacrificing dynamic performance. Elastomeric vitrimers, which are naturally flexible and prone to creep under stress, might benefit from a proportion of static cross-links that prevent deformation while retaining reprocessability and self-healing capabilities *via* dynamic linkages.^152–155^ Static and dynamic cross-links enable fine-tuning of the properties of the material, thus fit for use in coatings, adhesives, and composites. Some dynamic reactions might be useful in both catalyzed and non-catalyzed environments, therefore offering a great degree of manufacturing process and application flexibility. Made *via* the interaction of boronic acids and diols, boronic ester linkages show dynamic behavior under aqueous conditions and might be started by Lewis acids like boron trifluoride.^156,157^ When amines or organometallic compounds catalyze urethane bonds, which are often associated with polyurethanes, they may engage in reversible dissociation and reformation.^158^ Diels–Alder reactions provide a thermoreversible bond exchange mechanism that allows for fine control over reprocessability and thermal stability, making them appealing in high-performance applications.^159^ Aside from chemical problems, practical considerations such as processing and application temperature windows must be considered during vitrimer design. Bond dynamicity must match the thermomechanical requirements of the application since there is no universally acceptable behavior for dynamic bonds across all use cases. Researchers may modify vitrimer properties to suit application needs by addressing crucial challenges in bond selection, catalyst utilization, and network design, opening the way for improvements in sustainable polymer technology.

### 3.5 Vitrimerization with diverse nanofillers: fabrication of vitrimer nanocomposites

Nanofiller incorporation with vitrimers provides tremendous prospects for improving the material's mechanical, thermal, electrical, and other functional qualities. Vitrimerization, which relies on dynamic covalent bonding, is typically compatible with a diverse spectrum of nanofillers, including inorganic nanoparticles, carbon-based nanostructures, organic nanofillers, and polymer-based nanofillers.^86^ The success of this integration is determined by the interaction of the nanofillers with the vitrimer matrix, as well as the nanofillers' effect on the dynamic behavior of the vitrimer matrix. Vitrimers having dynamic covalent bonds can be chemically changed to interact with surface-functionalized nanofillers, such as silane-modified silica nanoparticles or graphene oxide functionalized with hydroxyl or carboxyl groups. This ensures strong interfacial connections, which improve material performance while maintaining the vitrimer's dynamic characteristics. Uniform nanofiller dispersion inside the vitrimer matrix is critical for improving composite characteristics. Strong interfacial contacts reduce agglomeration and increase stress transmission.^160–163^ Carbon nanotubes and graphene derivatives, when correctly disseminated, can strengthen the matrix while retaining its reprocessability and self-healing properties. Bond exchange mechanisms in vitrimers might be influenced by different types of nanofillers, such as clays or metal nanoparticles. Both catalysts and inhibitors affect material reprocessing behavior and stress relaxation. While some create steric hindrance, hence affecting bond dynamics, metallic nanofillers may speed up transesterification or disulfide exchange. Nanofillers such as silica nanoparticles and carbon-based nanostructures improve thermal stability, stiffness, and toughness in vitrimers, increasing the glass transition temperature and lowering thermal creep for high-performance applications.^72,83^ These enhancements retain the material's reprocessability thanks to dynamic covalent bonding. The addition of nanofillers to vitrimer precursors can enhance their viscosity, making processing difficult. The optimization of filler loading and dispersion methods is crucial for assuring processability. High filler loadings can restrict polymer chain mobility and also diminish dynamic bond exchange efficiency. Crucially, the design of nanofiller–matrix interactions preserves matrix flexibility. Some nanofillers may degrade over time or after numerous reprocessing cycles, reducing the vitrimer's long-term functioning.^164^ Vitrimerization's compatibility with varied nanofillers provides a flexible technique for generating multifunctional materials with specialized properties for advanced applications. By carefully selecting and developing nanofiller–matrix interactions, a compromise may be struck between the benefits of vitrimers and the additional functions of nanofillers, opening the way for novel materials with substantial industrial and technical value.

### 3.6 Fabrication techniques for vitrimer nanocomposite preparation

A wide range of novel approaches is utilized in fabrication technology to generate vitrimer nanocomposite materials with better physical and chemical qualities. Solution mixing, which generates composites by adding nanoparticles to monomer solutions, is one of the most basic ways. Surface modification methods like silane coupling are often employed to increase the dispersion and compatibility of nanomaterials with polymer matrices. For example, this method resulted in improved performance of cellulose-functionalized halloysite nanotubes (HNT-C) paired with epoxy oligomers.^89^ Waterborne vitrimers, which employ aqueous-based colloidal self-assembly to make long-lasting, recyclable nanocomposites with features like shape-locking and glueless lamination, are another intriguing topic.^93,165^ By employing heat to immobilize polymer networks, solvent-free processes like ball milling and hot pressing provide ecologically benign choices for dispersing nanoparticles and generating materials with good thermal characteristics and dimensional stability.^166,167^ Melt blending, also known as melt compounding, is an industrially scalable, solvent-free method of blending vitrimer resin and fillers at high temperatures to achieve effective dispersion by shear forces.^168–170^ In the presence of nanofillers, *in situ* polymerisation promotes the formation of polymer networks, which generally increases filler–matrix interactions, particularly when functionalised fillers are used.^171^ Another scalable, solvent-free method suitable for large-scale manufacturing is reactive extrusion, which combines polymerisation and melt processing. Particularly in films or coatings, intricate procedures like interfacial polymerisation and layer-by-layer assembly offer exact structural control.^136,172^ Furthermore, because vitrimer nanocomposites are reprocessable and self-healing, additive manufacturing methods like 3D printing are becoming more and more popular.^173,174^ The choice of production process depends on the type of filler, the intended end-use, and the matrix chemistry. To guarantee vitrimer performance and recyclability, processing parameters must be carefully controlled. When compared to randomly dispersed alternatives, aligned vitrimer nanocomposites, which are created utilizing procedures like uniaxial stretching or hot pressing, provide greater mechanical qualities.^175^ By creating chemical connections between nanoparticles and polymer matrices, reaction-based approaches further increase material performance. Examples include epoxy-functionalized silica in rubber matrices or diazo-coupling procedures to link carbon black or carbon nanotubes (CNTs) with natural rubber and carbon nanodots with ENR rubber. These technologies yield composites with outstanding self-healing, recyclability, and mechanical strength properties.^176–178^ Overall, the capacity to adjust processing settings or examine novel chemistries to customize material characteristics underscores the revolutionary relevance of fabrication technology in furthering material research.

## 4. Types of nanofillers used in vitrimers: their functions and impact on properties

### 4.1 Types of nanofillers

Various nanofillers have been used by researchers to enhance and reinforce the targeted property of the vitrimer composites. Such nanofillers are categorized into three major categories: inorganic nanofillers, carbon-based nanofillers, and miscellaneous hybrid nanofillers.

#### 4.1.1 Inorganic nanofillers (silica, alumina)

In particular, inorganic nanofillers are essential for improving the overall stability and performance of vitrimer matrices. The addition of various inorganic fillers, such as silica (SiO_2_), aluminum oxide (AlO_3_), and other metal oxides, to the dynamic covalent network of the vitrimer matrix significantly improves the mechanical strength, thermal stability, and dimensional integrity of the composite material. When the surface is functionalized to facilitate covalent bonding or to work with dynamic exchangeable groups, significant interactions can form at the interface with the polymer matrix due to the high surface area and nanoscale size of the fillers. Whether the filler surface slows down or participates in bond exchange reactions will determine how quickly or effectively these interactions assist in reducing stress.^179^ Functionalized inorganic fillers can also act as catalysts to accelerate dynamic bond rearrangement, which would facilitate self-repair and recycling of the material.^180^ Moreover, inorganic nanofillers can act as barriers against solvents, oxygen, and moisture to protect the matrix during mechanical or thermal cycling. However, the concentration and surface chemistry of reactive surfaces and heavy loading must be carefully controlled since they may impact network dynamics or create unwanted side reactions. For challenging applications, inorganic nanofillers provide a reliable method of creating vitrimer nanocomposites by striking a compromise between structural reinforcement and dynamic reprocessability.

**Fig. 3:**
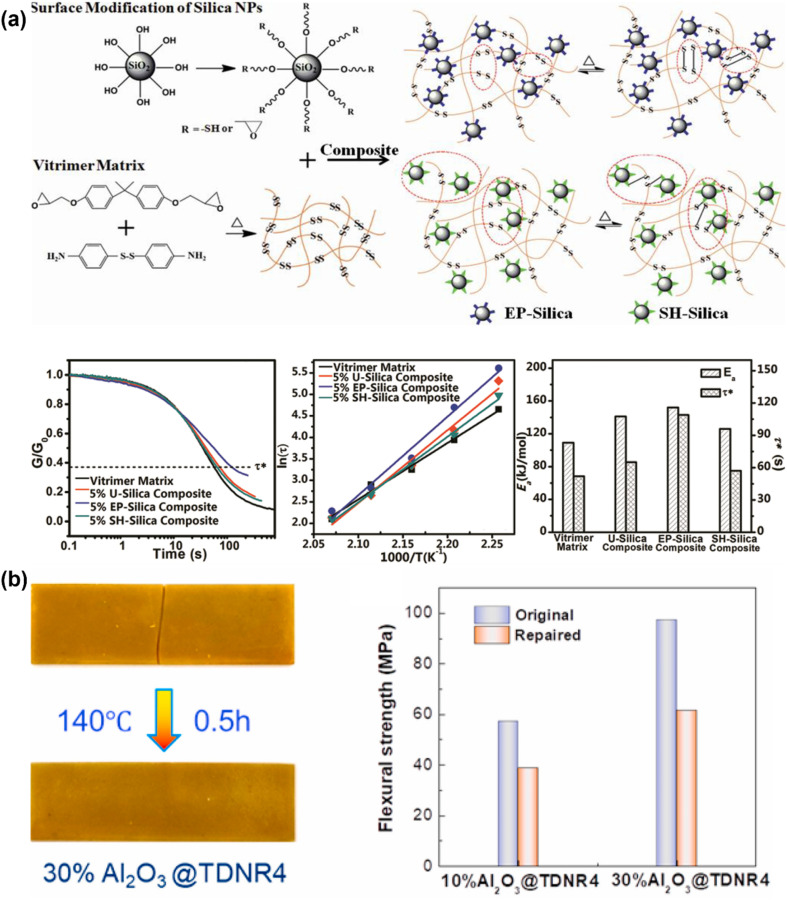
(a) Schematic representation of the synthesis, stress relaxation mechanism, and corresponding data of the epoxy-based vitrimer nanocomposite modified with the epoxy and thiol functionalized silica nanofiller. Reproduced with permission from ref. 184 Copyright 2020 Elsevier Ltd. (b) Fracture repairing and flexural strength of the original and repaired (with different contents of Al_2_O_3_) reinforced TDNR vitrimer nanocomposites. Reproduced with permission from ref. 186 Copyright 2021 Elsevier Ltd.

Typical fillers like silica (SiO_2_) or aluminum oxide (Al_2_O_3_) were used in vitrimer nanocomposites. Legrand *et al.* reported how silica nanoparticles (NPs) affected the mechanical and viscoelastic characteristics of epoxy-based vitrimer nanocomposites.^72^ The findings indicated that the vitrimer silica nanocomposites containing up to 40 wt% fillers were easily processed on a large scale without any solvent. Functionalized silica fillers enhance adhesion, dispersion, and mechanical properties by forming a covalent bond with the vitrimer matrix. Stress relaxation is also accelerated by surface exchangeable bonds compared to non-functionalized fillers. In a similar study for polyhydroxy urethane-based vitrimer nanocomposites, the dynamic exchanges due to the interaction of the modified silica filler surface and vitrimer matrix network are also examined.^181^ The vitrimer nanocomposite can restore its original characteristics by slowing stress relaxation with non-reactive nanofillers. However, introducing reactive nanoparticles into the matrix causes faster stress relaxation but also loss of initial properties due to side reactions between the vitrimer matrix and silica functionalities. With increased silica nanoparticle concentration in vitrimer nanocomposites, it exhibits varying cross-link density, creating a denser network, which reduces the stress relaxation time and macroscopic flow.^182^ Another study used vinylogous urethane vitrimers to dynamically cross-link surface-modified silica in the presence of Zn(ii) ions, which might significantly improve the mechanical characteristics of composites.^183^ Huang *et al.*^184^ described the development of a disulfide functional epoxy-based vitrimer matrix reinforced with functional silica nanoparticles, having thiol functionalities inserted on the silica nanoparticle surface. As a result, the functionalized nanoparticles provided enhanced mechanical characteristics and stress relaxation behavior in the reinforced vitrimer nanocomposite, which is shown in Fig. 3a. Furthermore, in comparison to non-functional nanocomposites, disulfide vitrimer epoxy/thiol silica nanoparticle composites demonstrated high self-healing efficiency in time-dependent healing tests with enhanced mechanical reinforcement.^89^ Spiesschaert *et al.* reported that an effective way to modify the viscoelastic characteristics of polydimethylsiloxane (PDMS) vinylogous urethane vitrimers is to add fillers based on silica or aluminum oxide.^185^ The study discovered that the material characteristics of reinforced vitrimers are directly impacted by the surface functionality of additional fillers. In contrast to neutral Al_2_O_3_, materials reinforced with acidic or basic Al_2_O_3_ had enhanced Young's modulus and lowered elongation. The investigation also revealed that switching between the Al_2_O_3_ varieties might somewhat alter the creep resistance and relaxation time. In order to create vitrimer composites based on a phenolic resin matrix with dynamic urethane linkages that encourage transcarbamoylation reactions, Liu *et al.* used Al_2_O_3_ particles. Because of the hydroxyl groups on their surface and their attraction for the isocyanates in the cross-linking agent, Al_2_O_3_ particles were able to disperse widely. Although only 60% of its initial flexural strength could be regained after breaking and hot pressing (140 °C, 30 min), different amounts of Al_2_O_3_ enhanced both the breaking and flexural strengths (Fig. 3b). The irreversible covalent connections that formed between the Al_2_O_3_ particles and the released isocyanates during the dynamic exchange are most likely the reason that it did not fully recover. Therefore, depending on the chosen chemistry, Al_2_O_3_ particles may potentially adversely affect the dynamic characteristics of vitrimers.^186^

#### 4.1.2 Carbon-based nanofillers: carbon nanotubes, graphene, and carbon nanodots

##### 4.1.2.1 Carbon nanotubes as a nanofiller

**Fig. 4:**
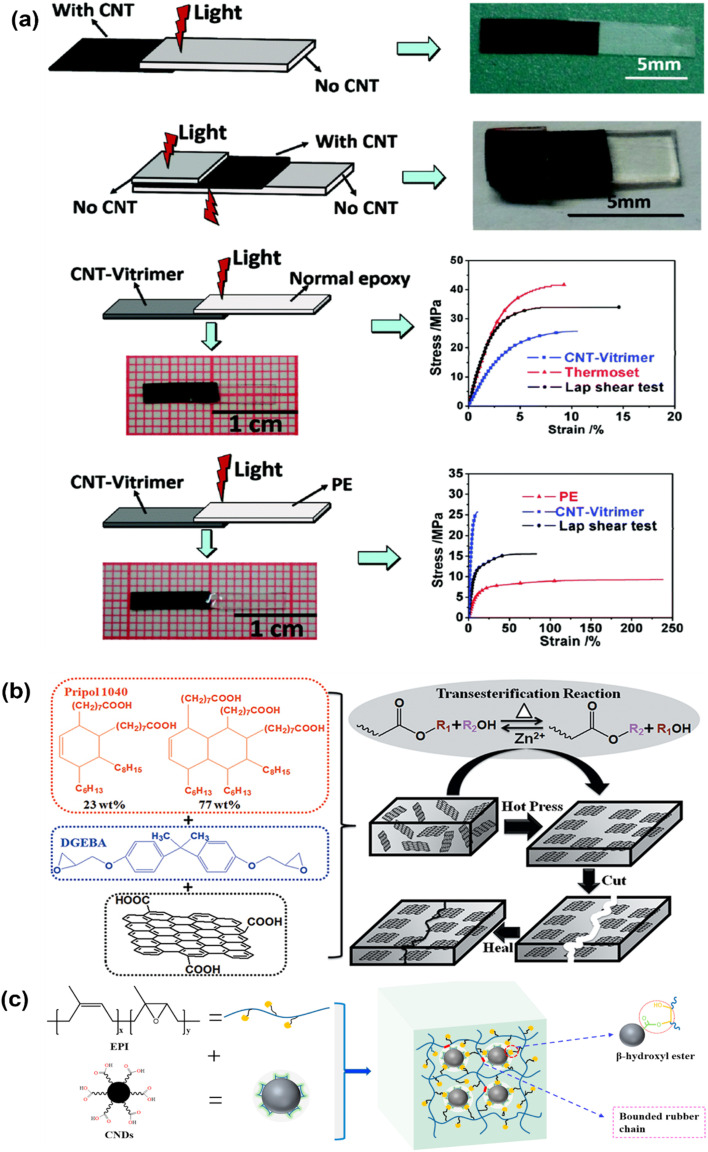
(a) Transmission welding and its corresponding data for welding with other materials of vitrimer nanocomposites with CNT and without CNT. Reproduced with permission from ref. 193 Copyright 2014 The Royal Society of Chemistry. (b) Chemical structure of monomers and the synthesis process of aligned and healable graphene/epoxy nanocomposites. Reproduced with permission from ref. 205 Copyright 2019 Frontiers in Chemistry. (c) Schematic representation of a dynamic reinforced network formation. Reproduced with permission from ref. 207 Copyright 2021 Elsevier Ltd.

The peculiar one-dimensional cylindrical nanocarbon composition and sp^2^ hybridization in the nanostructure of carbon nanotubes make them an effective nanofiller for polymers.^187,188^ Carbon nanotubes in both single-walled and multi-walled forms have been generated regularly.^189^ Despite having advantageous functional properties like thermal conductivity, tensile strength, and electrical conductivity, carbon nanotube-reinforced polymeric nanocomposites are prone to aggregation in polymeric matrices due to their large surface area and van der Waals interactions.^190^ CNTs absorb light at nearly every wavelength and convert it to heat, producing quick and accurate local heating that may be very helpful for vitrimer matrix composites. Since CNT aggregation can result in notable changes to material characteristics, adequate processing methods and loading concentration are necessary to support insufficient dispersion and technical performance to handle this aggregation problem.^191^ Modifications to carbon nanotubes may also improve their interactions and dispersion with polymer chains.^192^ For instance, Yang *et al.*^193^ brought up the difficulty of assembling traditional epoxy materials by welding them together since epoxies are not soluble or meltable. They offered a straightforward yet incredibly effective method by investigating the photothermal impact of carbon nanotubes (CNTs) to control the transesterification reaction in vitrimers. To create the epoxy vitrimer with 1 wt% CNT, the diglycidyl ether of bisphenol A (DGEBA) resin and adipic acid were reacted in the presence of a triazobicyclodecene transesterification catalyst. The light could fuse the resultant epoxy-based vitrimer reinforced with CNT in just a few minutes. CNT vitrimers might be welded with various epoxy or thermoplastic polymers *via* gearbox welding, which is impossible with direct heating. As a result, CNT vitrimer composites were effectively fused with non-CNT vitrimers using infrared laser irradiation (Fig. 4a). According to their findings, it offers a very effective technique that uses the photothermal effect of carbon nanotubes (CNT) to initiate transesterification processes in epoxy-based vitrimers allowing for quick and flexible light-driven welding and healing. Other epoxies and thermoplastics, as well as materials with different chemical compositions and physical characteristics, can be joined by CNT-dispersed vitrimer epoxies in a matter of seconds to minutes. Without the need for glues or molds, this method enables gearbox welding for intricate curves or *in situ* repairs of delicate, valuable artifacts, in contrast to conventional heating or currently used photo-weldable networks. Because of their extensive light absorption, the CNTs may be used with a variety of light sources outside of infrared, and their capacity to transform electric or magnetic energy into heat creates opportunities for alternate welding and healing methods. This reliable, easy-to-use, and scalable process shows remarkable promise for advanced applications in contemporary technology and mass manufacturing.^193^ To aid in transesterification and endow vitrimers with improved electrical conductivity, CNTs were also added to conductive polymers. It should be noted that typical vitrimers' electrical characteristics and transesterification rates are insufficient for a variety of real-world uses. Stress relaxation was used to assess the performance of the transesterification process, and after doping with just 3 wt% CNT/polypyrrole (PPy), the relaxation rate was 3.6 times quicker. The interfacial contact between CNT/PPy and the vitrimer matrix, as well as the higher thermal conductivity of CNTs, contributed to the enhanced transesterification in stress relaxation. Pure CNTs as dopants produced minimal enhancement due to substantial matrix agglomeration, in contrast to CNT/PPy vitrimer matrix composites. Furthermore, the conductivity was enhanced by several orders of magnitude using CNT/PPy doping.^194^ In addition to having outstanding electrical conductivity and mechanical flexibility that permits bending, stretching, rehealing, and closed-loop recycling, composites made of polyimine vitrimer matrices and multiwalled carbon nanotube (MWCNT) fillers also display dynamic covalent bond exchange. Less than 10 wt% MWCNTs were dispersed in a solution of terephthalaldehyde, diethylenetriamine, and the cross-linker tris(2-aminoethyl)amine to create these composites. These materials achieve around 97% conductivity recovery and 100% component reuse, hence lowering manufacturing costs and electronic waste. They also retain their mechanical and conductivity qualities even after being repeatedly reshaped, repaired, or recycled. Together with the conductive qualities of CNTs, these composites' heat-driven malleability makes them perfect for complicated geometries, flexible electronics, and *in situ* repairs.^84^ To sum up, CNT–vitrimer interactions are a combination of physical interactions (π–π stacking, hydrogen bonding), covalent bonding (by functionalized groups), and stimuli-responsiveness (photothermal/electrothermal effects). Because of these synergistic effects, CNTs are positioned as mechanical reinforcers and active components that can modify the kinetics and efficiency of vitrimer chemistry. This makes it possible for next-generation nanocomposites to have improved functionality, including programmed welding, healing, and reshaping.

##### 4.1.2.2 Graphene as a nanofiller

Graphene is a one-of-a-kind two-dimensional nanosheet composed of carbon atoms that were created through sp^2^ hybridization. Excellent mechanical, electrical, and optical qualities are added, significantly improving the performance of polymer matrices.^195,196^ In addition to its readily available manufacturing processes, including chemical vapor deposition, mechanical exfoliation of graphite, and other chemical or laser-based procedures, graphene is unique among nanocarbons because of its exceptional structural and physical properties.^197,198^ Graphene has outstanding mechanical strength (with Young's modulus of around 1 TPa), remarkable thermal conductivity (∼3000–5000 W mK^−1^), and tremendous electron mobility (∼200 000 cm^2^ V^−1^ s^−1^). Graphene's fragile structure may result in problems like van der Waals force-induced aggregation or wrinkling. These issues can be addressed by chemical changes or oxidation, making graphene a viable nanofiller for polymer systems.^199–201^ The structure of common thermosetting polymers has high covalent crosslinking, which makes recycling and reprocessing challenging.^202^ This has led to the development of novel materials, such as vitrimer resins, which are recyclable, processable, and biodegradable.^203,204^ Carbon-based nanoparticles, such as graphene, have been shown to have superior reprocessing and recycling capabilities, as well as improved physical characteristics and shape recovery in vitrimer nanocomposites. Furthermore, these nanocomposites have exceptional self-healing properties, increasing the possible use range. Chen *et al.* used the dynamic nature of epoxy vitrimers to create aligned graphene nanoplate (GnP)/epoxy composites in a simple and scalable hot press process. Because of graphite's 2D structure and volume exclusion effect, the bond exchange and topological rearrangement related to the viscous flow of the epoxy vitrimer during the hot pressing procedure permitted spontaneous orientation of GnP in the vitrimer matrix (Fig. 4b). SEM imaging confirms that the spontaneous orientation of GnPs during hot pressing is made possible by the epoxy vitrimer's dynamic bond exchange and viscous flow features. At ideal compression ratios, tensile tests show strength increases of up to 173.3% compared to pristine epoxy, demonstrating how much this alignment improves mechanical qualities. In addition, the vitrimer matrix provides exceptional recyclability and healability, retaining virtually full strength in recycled samples and recovering over 95% of its mechanical qualities after cutting. This study demonstrates the transformational potential of vitrimer-based composites for applications in lightweight, high-strength, and sustainable materials, especially when combined with the remarkable conductivity, flexibility, and closed-loop recyclability seen in prior vitrimer–carbon nanotube systems. These discoveries pave the way for large-scale manufacturing of aligned thermosetting composites with specialized uses and favorable environmental effects.^205^ Graphene was added to styrene-butadiene rubber (SBR) to improve its mechanical qualities, malleability, and multi-stimuli response. Without a catalyst, the crosslinked networks could change their topologies through transimination processes in the bulk network and the graphene interphase, which allowed them to be regenerated and reshaped when heated or exposed to infrared radiation. The mechanical characteristics of vitrimer composites were enhanced by the addition of graphene to the SBR network.^206^ Additionally, following many recycling generations (cut and hot-pressed), the mechanical characteristics of the samples (with varying graphene percentages) were nearly the same as those of the initial reprocessed samples. Using self-healing molecular dynamics simulations between a graphene oxide (GO)/vitrimer nanocomposite and pristine vitrimers, graphene's boosting impact was recently verified.^74^ DGEBA epoxy with 2-AFD as the hardener served as the foundation for the simulations. The findings showed that the *T*_g_ of vitrimers was lowered by the addition of GO. In line with a prior paper on GO vitrimers, the nanocomposite's self-healing capabilities were also superior to vitrimers across all temperature ranges.^83^ Additionally, atomistic studies showed that GO/vitrimer nanocomposites had a higher number of new disulfide bonds that exchanged during the self-healing simulation, confirming that the bond exchange reaction was accelerated by the addition of GO to the vitrimer. These simulation results suggest that other nanofillers might be used for the same objective, and it's noteworthy to note that the *T*_g_ decrease in polymeric nanocomposites is a generic phenomenon seen in different filler/matrix compositions.

### 4.2 Carbon nanodots as a nanofiller

Carbon nanodots were also utilized as nanofillers to create vitrimer nanocomposites. The dynamic network with exchangeable β-hydroxyl ester bonds was constructed by Niu *et al.* using carboxylated carbon nanodots (CNDs, carbon nanoparticles smaller than 10 nm in size) and epoxidized polyisoprene (EPI) as a cross-linker and reinforcement. The mechanical characteristics and dispersion of CNDs in the rubber matrix were enhanced by these dynamic interactions at the interface between EPI and CNDs (Fig. 4c). The CND vitrimer composite had good shape memory performance and was able to be reshaped and reprocessed.^207^

#### 4.2.1 Cellulose-based nanofillers

**Fig. 5:**
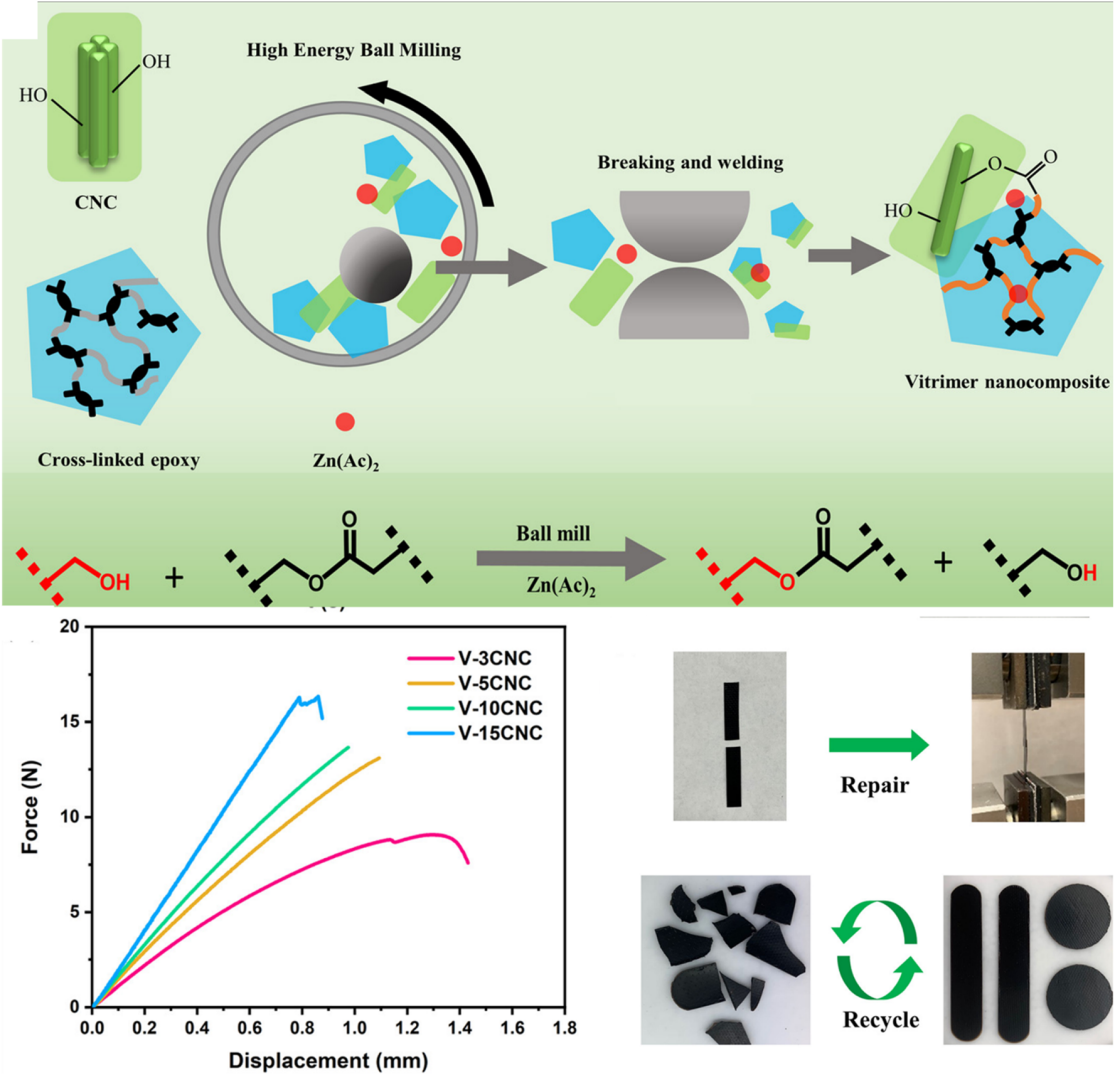
Schematic representation of cross-linked epoxy vitrimerization using CNCs with force–displacement curves (lap-shear test), repairing and recycling of broken epoxy/CNC nanocomposites. Reproduced with permission from ref. 167 Copyright 2021 American Chemical Society.

Cellulose nanocrystals (CNCs) are the crystalline areas that are separated from cellulose microfibrils, primarily owing to intense acid hydrolysis at high temperatures. With their high aspect ratio, large surface area, and great mechanical strength, CNCs are inexpensive, environmentally benign, and sustainable materials that may be used in a variety of applications. Recently, a unique idea known as “vitrimerization,” which involves transforming thermoset polymer networks that are irreversibly crosslinked into dynamic exchangeable networks, was used in these CNCs. The concept uses a planetary ball mill to crush thermosets mechanochemically with a catalyst to facilitate recycling and reprocessing. The initially irreversibly crosslinked network becomes a vitrimer when the hydroxyl functions at the interface accomplish a suitable dynamic exchange. Yue *et al.* showed that adding CNCs as a source of external hydroxyl groups to the mechanochemical vitrimerization process might enhance both the epoxy vitrimer's thermomechanical characteristics and exchange reaction rate.^167^ Following processing, the new epoxy vitrimer showed typical vitrimer polymer characteristics, including a reorganization of the network structure. The welding caused by the transesterification exchange reaction is the foundation for network reformation and property recovery. Apart from the improved transesterification exchange processes, the bio-based CNCs enabled mechanical repair and recycling as well as improved thermomechanical characteristics of the nanocomposites (Fig. 5). This process allows the creation of vitrimer polymers from thermoset wastes rather than synthesizing recyclable vitrimers, and it may be appropriate for industrial uses.^167^ In a similar study, Gao *et al.* created a vitrimer nanocomposite crosslinked with epoxidized soybean oil (ESO) and reinforced with carboxyl-functionalized cellulose nanocrystals (CNCs).^208^ Carboxylated nitrile butadiene rubber (XNBR) served as the foundation for the nanocomposite. The ESO functioned as a dynamic crosslinker by combining with the carboxylated CNCs and the XNBR matrix to form β-hydroxy ester linkages, which encourage ester–hydroxyl bond exchange reactions in XNBR vitrimer nanocomposite systems. These dynamic covalent bonds enabled the remarkable shape memory behaviour and recyclability of the resulting nanocomposites, underscoring the complementary functions of bio-based crosslinkers and surface-modified nanofillers in enhancing the functional performance of rubber-based vitrimer materials.

Due to their high aspect ratio, strength, and natural breakdown, cellulose nanofibers (CNFs) are also gaining a lot of interest as eco-friendly nanofillers. However, because they cluster together during static curing procedures, they frequently don't mix well when added to crosslinked polymer matrices. A recent study by Ran *et al.* came up with a new way to make CNF-reinforced epoxy vitrimer composites using dynamic crosslinking.^209^ To improve the distribution of the nanofillers, this technique makes use of vitrimer chemistry. CNFs were initially combined with the catalyst, curing agent, and epoxy monomer in this process. The network then underwent a dynamic curing procedure that allowed it to change shape in real time and more evenly distributed the filler. The CNF distribution in the epoxy vitrimer/CNF composites produced by this technique was consistent, and there was no noticeable aggregation at loadings up to 0.75 weight percent. Compared to static curing methods, this is a significant improvement. At this optimal concentration, the composite's tensile strength increased 2.26 times, and its Young's modulus increased 3.61 times. This demonstrates unequivocally how effectively CNFs may strengthen materials in dynamic environments. This study indicates that filler aggregation issues in vitrimer systems may be effectively addressed by dynamic covalent network creation. It also makes it possible to use this technique with vitrimer matrices and other bio-based nanofillers.

#### 4.2.2 Other nanofillers for the vitrimer matrix

**Fig. 6:**
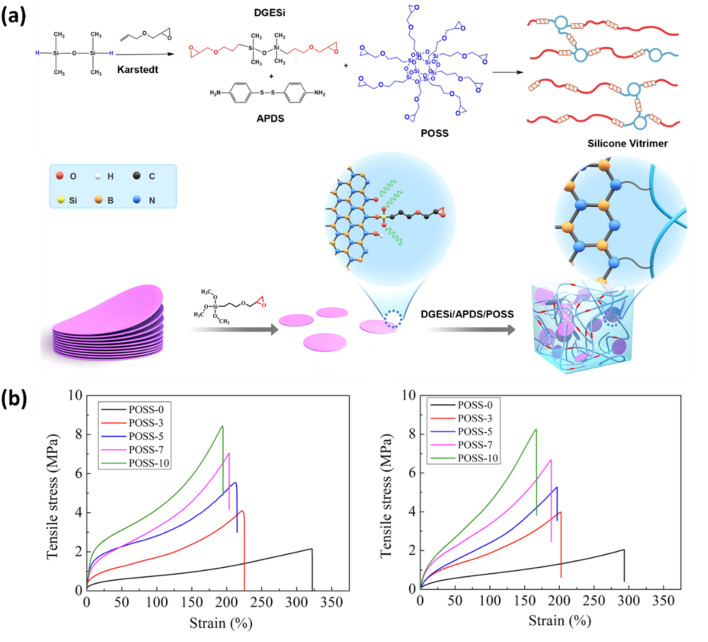
(a)Schematic diagram for the preparation of the silicone vitrimer matrix and nanocomposite, (b) stress–strain curve for before and after healing of the silicone vitrimer reinforced with different POSS contents. Reproduced with permission from ref. 211 Copyright 2022 Elsevier Inc.

Polyhedral oligomeric silsesquioxane (POSS) nanostructures are extremely adaptable substances that find utility in everything from aerospace to biomedicine. In polymer nanocomposites, they serve as reinforcing agents, improving characteristics like stiffness, resistance to radiation and high temperatures, and decreased mass density. The structure of the siloxane (Si–O–Si) cage nanostructure that POSS has allows for the incorporation of functional groups. POSS in catalyst-free vitrimers based on vinylogous urethane chemistry with a high biosourced component was investigated by Hajiali *et al.*^210^ In comparison to the unmodified vitrimer, they found gains in tensile modulus, strength, and thermal degradation temperature by adding NH_2_-functionalized POSS at different loadings. POSS is a vital nanofiller of silicon elastomer vitrimer nanocomposites, which have been the topic of recent studies on thermal conductivity. Functionalized boron nitride nanosheets (fBNNS) were utilized to boost thermal conductivity and octaglycidyl POSS to improve the mechanical properties of a silicon vitrimer cross-linked with 4-aminophenyl disulfide (4AFD) (Fig. 6a and b). Even after six healing cycles, the addition of 66 wt% fBNNS had no harmful effect on thermal conductivity despite considerably lowering the material's repair efficiency. These compounds have promise for application as electronic device thermal interfaces.^211^ Another recent study by Quinteros-Sedano *et al.*^212^ described a novel method using thermoreversible organic nanofillers (TRONs), which are low-molecular-weight substances that act as plasticizers at processing temperatures and as reinforcing fillers at service temperatures. This method is demonstrated using two dibenzylidene sorbitol derivatives as TRONs and an elastomeric polybutadiene vitrimer based on dioxaborolane as the matrix. According to the study, TRON-loaded vitrimers reduce viscosity at processing temperatures while increasing creep resistance and tensile properties at service temperatures.^212^

### 4.3 Nanofillers: transforming vitrimer nanocomposite performance

The functional characteristics of vitrimers are significantly enhanced by nanofiller reinforcement, which improves their rheological, mechanical, thermal, self-healing, and other properties. Vitrimers are made more versatile for various applications by the incorporation of nanofillers such as silica, graphene, carbon nanotubes, and cellulose nanofillers, which greatly increase the mechanical strength, toughness, and fracture resistance of the vitrimer nanocomposite. Furthermore, nanofillers enhance recyclability and self-healing efficiency through dynamic bond exchanges. They also improve the rheological behavior of the vitrimer, which makes industrial applications more processable. Nanofiller reinforcement provided more advanced functionalities such as improved fire resistance, reduced gas permeability for better barrier properties, and increased electrical conductivity, widening the usage of vitrimers in high-performance and multifunctional materials.

#### 4.3.1 Effect of nanofillers on the mechanical properties of vitrimers

**Fig. 7:**
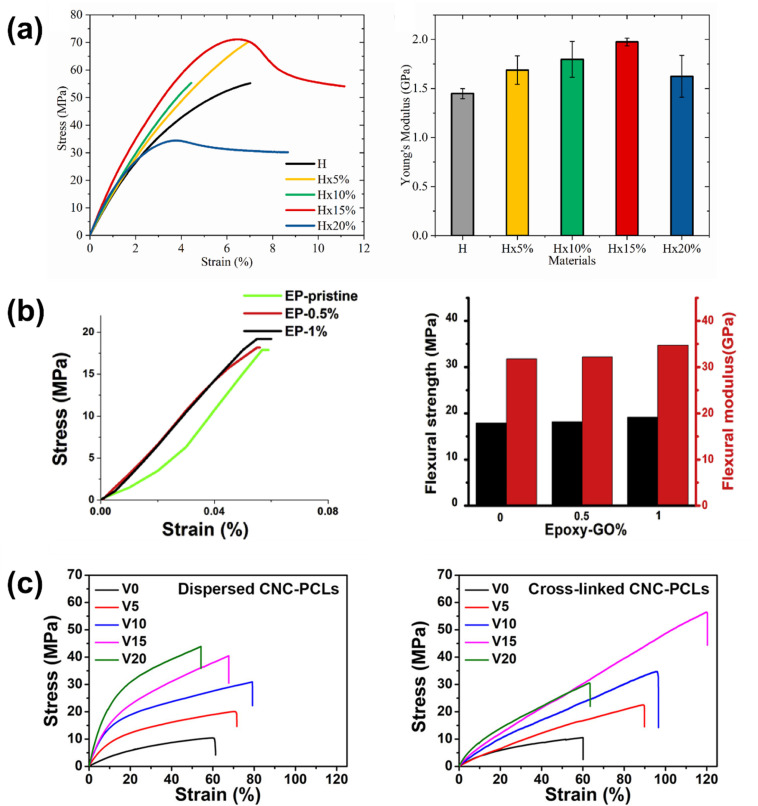
(a) Stress–strain and Young's modulus curves of silica–epoxy vitrimer nanocomposites with different weight loadings of nanoparticles. Reproduced with permission from ref. 219 Copyright 2022 Elsevier Ltd. (b) Stress–strain, flexural strength, and flexural modulus relationship between different epoxy vitrimer nanocomposites. Reproduced with permission from ref. 83 Copyright 2022 Elsevier Ltd. (c) The vitrimer composites' strain–stress curves before and after hot-pressing at 1 MPa for 24 hours at 160 °C. Reproduced with permission from ref. 221 Copyright 2023 American Chemical Society.

The role of nanofiller reinforcement in the vitrimer matrix is vital, as it increases the mechanical strength and toughness of the matrix. Nanofiller addition also increases the load transfer capability of the matrix at the molecular level. Silica, carbon nanotubes, graphene oxide, cellulose nanocrystals, and other nanofillers are mixed into the vitrimer matrix to create a more rigid and robust hybrid structure.^167,213–216^ These nanofillers restrict crack propagation, stress distribution over mechanical load, and energy dissipation, resulting in higher elasticity, fracture toughness, and tensile strength in the vitrimer matrix.^217,218^ Furthermore, interfacial interactions of the nanofiller with the vitrimer matrix improve the dynamic bond exchange mechanism in vitrimer networks, which enhances the mechanical performance while maintaining self-healing and recyclability. Chen *et al.* have reported an epoxy-based vitrimer reinforced with silica nanoparticles, which significantly improves the tensile characteristics of the vitrimer matrix. The tensile stress–strain curve showed that with the increase in nanoparticle loadings from 5 to 15 wt%, Young's modulus increased from 1.7 to 2.0 GPa and tensile stress from 71.8 to 72.4 MPa (Fig. 7a). Meanwhile, for the virgin epoxy vitrimer, the modulus was 1.4 GPa. However, at 20 wt% loading of nanoparticles, Young's modulus falls to 1.6 GPa due to the generation of defects such as voids and micro-cracks, as well as decreased cross-linking produced by nanoparticle–anhydride interactions. However, the modulus at 20 wt% remains more significant than that of the virgin material.^219^ In another study, Barabanova *et al.* reported a silica nanofiller-reinforced epoxy-based thermoset vitrimer, which was synthesized utilizing the diglycidyl ether of bisphenol A (DGEBA), 4-methylhexahydrophthalic anhydride (MHHPA) as a hardener, zinc acetylacetonate as a transesterification catalyst, and 10–15 nm silica nanoparticles, as a nanofiller. The addition of 5–10 weight percent silica nanoparticles significantly improved the material's tensile stress by 25%, the elastic modulus by 44%, and the dimensional stability by reducing thermal expansion. These increases were confirmed by tensile and thermomechanical testing, while the zinc acetylacetonate catalyst allowed chain exchange operations and provided welding capabilities upon heating.^213^ Graphene nanofillers also enhanced the mechanical strength and toughness of epoxy vitrimer matrices. Numerous studies suggest that adding graphene, in the form of graphene oxide (GO) and graphene nanoplatelets (GNP), increases overall mechanical performance and the tensile and flexural strengths of vitrimer nanocomposites. Vashchuk *et al.* have synthesized an epoxy-based vitrimer nanocomposite using a thiol–epoxy click reaction with ≤1.0 wt% graphene oxide (GO). This integration resulted in significant improvements in mechanical and thermal characteristics. At 0.5 wt% GO, tensile strength and Young's modulus rose by 69% and 46%, respectively, while 1.0 wt% GO greatly enhanced ductility, increasing it almost fourfold.^220^ In a similar study, Krishnakumar *et al.* developed vitrimeric features by disulfide exchange in an epoxy vitrimer network where the addition of 1 wt% graphene oxide (GO) decreased the glass transition temperature, enabling low-temperature self-healing and shape memory capabilities. As shown in Fig. 7b, the resultant nanocomposite, EP-1%, demonstrated 7.1% and 9.4% greater flexural strength and modulus, respectively, compared to pure epoxy vitrimers.^83^ The mechanical strength and toughness of vitrimers are greatly increased by the addition of cellulose nanofillers. Numerous studies show that cellulose nanofibers (CNFs) and cellulose nanocrystals (CNCs) enhance the characteristics of polymer composites by means of structural reinforcement and interfacial compatibility. Sun *et al.*^221^ reported a vitrimer matrix of epoxy-thiol covalent adaptive networks (CANs) cross-linked with core–shell CNC–PCL (polycaprolactone) nanohybrids by epoxy-thiol “click” reactions and a hot-pressing transesterification procedure was used to create the new nanocomposite. The CNC–PCL nanohybrids greatly improved mechanical characteristics while efficiently controlling stress relaxation and the transesterification activation energy. When compared to unfilled vitrimers, the composite showed 2.5 times better Young's modulus, 5.4 times better fracture stress, and 2 times better fracture strain (Fig. 7c).^221^ The mechanical performance of vitrimer matrices can be greatly improved by adding nanofillers, such as cellulose, graphene, and silica, as these studies clearly show. To increase tensile strength, modulus, and ductility, surface compatibility and optimal filler loading are essential. However, a high filler component might compromise the integrity of the material by causing aggregation or microstructural flaws. In general, the strategic use of nanofiller reinforcement to modify vitrimer characteristics for high-performance and multipurpose applications is revealed.

#### 4.3.2 Nanofillers enhancing the thermal stability and conductivity of vitrimers

The inclusion of nanofillers significantly enhances the thermal properties and conductivity of vitrimers, making them suitable for complex applications. Better thermal management is made possible by vitrimers, which are characterized by their dynamic covalent bonding, and the strategic use of nanofillers, which enhance material performance and facilitate heat transmission. The addition of nanofillers to vitrimers enhances thermal conductivity and yields better thermal performance than traditional thermosetting polymers by improving alignment and creating efficient heat pathways. Additionally, it enables the recycling and reprocessing of materials.^55^ The use of nanoparticle fillers improves the thermal properties and conductivity of epoxy vitrimers. This enhancement depends on a number of elements, including the kind of filler, aspect ratio, filler loading, degree of dispersion, and the contact between fillers and polymers.^222^ The integration of thermally conductive nanofillers into vitrimer matrices has proven to be an efficient technique for boosting both thermal conductivity and thermal stability, which are crucial for applications in electronics, energy storage, and thermal interface materials (TIMs). Among these, multi-walled carbon nanotubes (MWCNTs), boron nitride (BN), and graphene oxide (GO) have emerged as promising prospects.

**Fig. 8:**
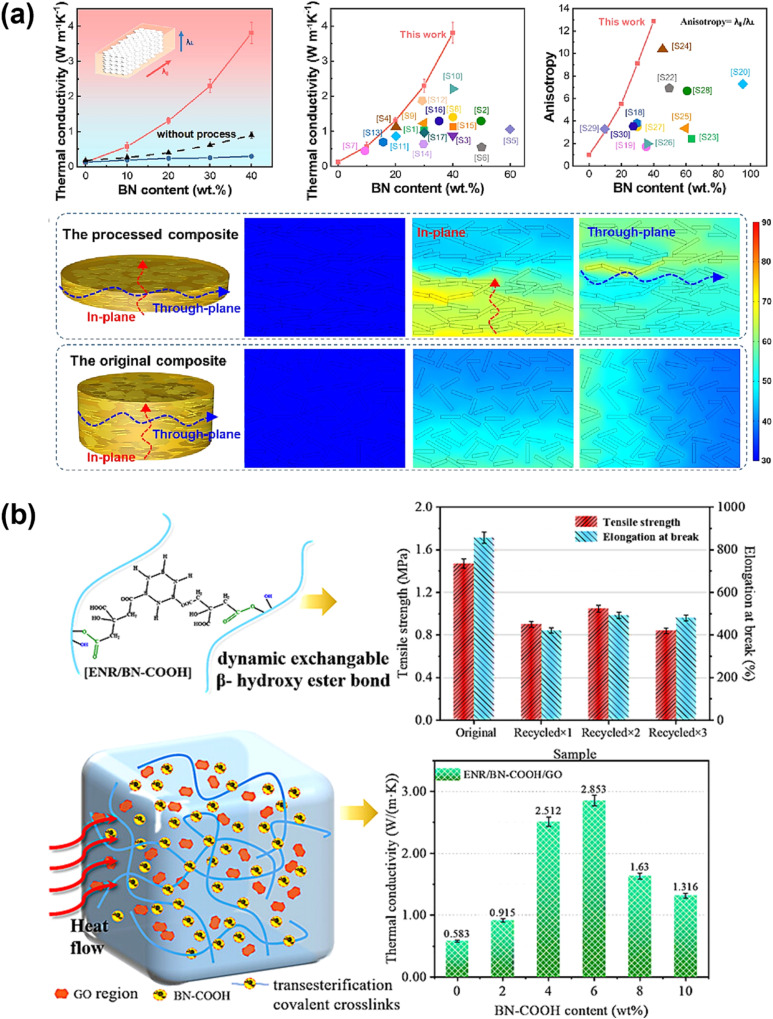
(a) The thermal conductivity of epoxy/BN composites in the parallel and perpendicular directions, comparison of epoxy/BN composites' thermal conductivity, anisotropy in the parallel and perpendicular directions, and simulation results between the original and treated composites with data from the literature. Reproduced with permission from ref. 224 Copyright 2023 Elsevier B.V. (b) The dynamic β-hydroxyl ester bonds formed between ENR and BN-COOH significantly improved the mechanical properties of the original and recycled nanocomposite and the effect of GO BN-COOH in the thermal conductivity of the vitrimer nanocomposite. Reproduced with permission from ref. 225 Copyright 2023 American Chemical Society.

Feng *et al.* used multi-walled carbon nanotubes as a nanofiller in an epoxy vitrimer matrix to improve the thermal conductivity of the vitrimer nanocomposite. The study demonstrated that polydopamine-coated MWCNTs enhance thermal stability and conductivity in epoxy vitrimers by improving interfacial interactions, resulting in better dispersion and fewer defects, thereby significantly increasing the thermal conductivity and mechanical properties of the composites.^223^ Liu *et al.* reported a high-performance vitrimer nanocomposite that was created by mixing boron nitride (BN) with an epoxy vitrimer that features a topologically multi-dynamic cross-linking structure as a matrix. By aligning BN nanofillers into a lamellar structure by hot pressing, interfacial defects were minimized by stress relaxation generated by disulfide bonds at 200 °C. High in-plane thermal conductivity (3.85 W m^−1^ K^−1^) with an anisotropy of about 13, as well as remarkable mechanical and thermal stability (R800 of 62.0%) and tensile strength (46.3 MPa), were all achieved by the resulting epoxy/BN (40 wt%) nanocomposite. The nanocomposite's ability to adapt to rough surfaces due to its vitrimeric nature makes it excellent for thermal interface materials (TIM) (Fig. 8a). When compared to a commercial silicone-based counterpart, the TIM device that employed this combination demonstrated excellent cooling performance, dropping the core temperature by 20 °C.^224^ Gong *et al.* used epoxidized natural rubber (ENR) and carboxylated boron nitride (BN-COOH) that were dynamically cross-linked to create recyclable and self-healable ENR/BN-COOH/GO nanocomposites, with graphene oxide (GO) serving as a reinforcing element. By increasing interfacial contact and energy dissipation, the dynamic β-hydroxyl ester bonds formed between ENR and BN-COOH significantly improved mechanical properties by 116% when compared to ENR and 79% when compared to ENR/GO (Fig. 8b). Transesterification procedures were made possible by these interchangeable links, which also offered recycling and self-healing properties. The development of effective thermal routes and a hybrid conductive network of BN-COOH and GO also allowed the nanocomposites to exhibit good thermal conductivity (2.853 W m^−1^ K^−1^ at 6 wt% BN-COOH). High thermal conductivity was maintained in recycled samples, demonstrating the thermal network's robustness.^225^

These investigations demonstrate how thermally conductive nanofillers, like graphene oxide, boron nitride, and MWCNTs, can improve the mechanical robustness and thermal control of vitrimer nanocomposites. Defect minimisation and effective heat transport are greatly aided by improved interfacial interactions, which are made possible by surface functionalisation or structural alignment. Furthermore, improved thermal conductivity is maintained even after recycling and reshaping thanks to the dynamic covalent chemistry of vitrimer matrices. Together, our results highlight the potential of vitrimer nanocomposites as cutting-edge, recyclable thermal interface materials (TIMs) for high-performance uses in energy and electronics systems.

#### 4.3.3 Role of nanofillers: self-healing and recyclability enhancement of vitrimers

**Fig. 9:**
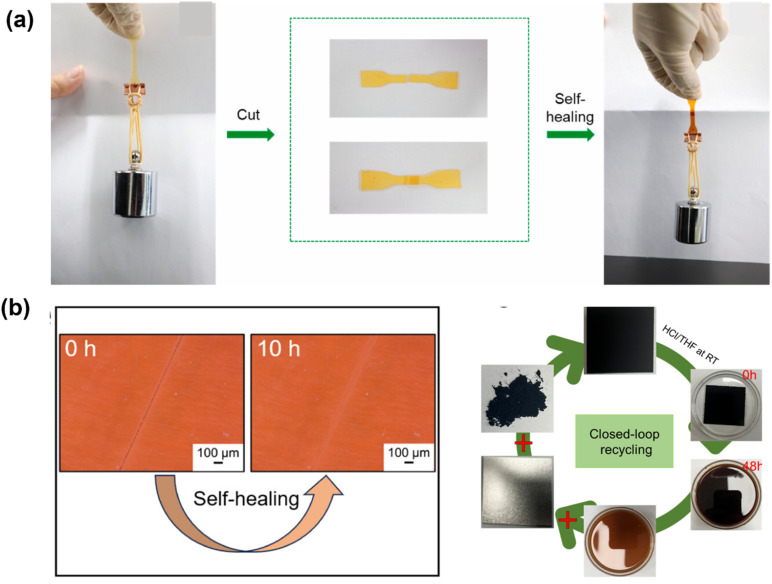
(a) Different self-healing tests for the silicone vitrimer matrix nanocomposite. Reproduced with permission from ref. 164 Copyright 2022 Elsevier Ltd. (b) Self-healing and closed-loop recyclability of an MWCNT reinforced epoxidized soybean oil-based vitrimer nanocomposite. Reproduced with permission from ref. 226 Copyright 2024 American Chemical Society.

The incorporation of nanofillers into vitrimers significantly improves the properties of self-healing and recyclability, establishing vitrimers as a potentially helpful option for the creation of sustainable materials. The addition of nanofillers such as graphene oxide and carbon nanotubes can improve the reprocessing and self-repair capabilities of vitrimers, which are distinguished by dynamic covalent bonding. Vitrimers also allow for structural change. The methodology and benefits of these developments are discussed in further detail in the following sections. Wang *et al.* developed a silicone vitrimer with the reinforcement of cellulose nanocrystals (CNC) and optimized molecular weight of cross-linker β-keto acid ester. Amino-functionalized CNC (M-CNC) was created by combining 3-aminopropyltriethoxysilane with a silicone rubber matrix that included amino side chains. The interaction of M-CNC, rubber chain amino groups, and cross-linker β-keto acid ester resulted in dynamic vinylogous urethane interfacial bonds. As a result, the mechanical characteristics, self-healing properties, and reprocessability of the vitrimer nanocomposite were significantly improved. After being laminated and heated at 150 °C for 30 minutes, the broken sample strips formed a strong connection that could support a 200 g weight (Fig. 9a). Tensile testing revealed that the repaired samples broke at new places instead of the interface, indicating that bond exchange and cross-linking were effective. The quick exchange of vinylogous urethane bonds is responsible for these self-healing properties, indicating the strong and long-lasting repair capabilities of vitrimer nanocomposites. Excellent recyclability was demonstrated by the successful reprocessing of crushed samples by hot-pressing them for one hour at 180 °C and 15 MPa, which produced a smooth surface and preserved their mechanical qualities. However, the elongation at break recovered to 96%, and the mechanical strength recovery was only 52% for samples cross-linked with small molecules. This provides insight into how the cross-linked structure affects mechanical property restoration and recyclability effectiveness.^164^ Wang *et al.* reported an epoxidized soybean oil-based vitrimer reinforced with multi-walled carbon nanotubes (MWCTs) to generate a biobased photothermal superhydrophobic coating with self-healing and closed-loop recyclability (Fig. 9b). In this work, they demonstrated that dynamic imine bonds provide superior closed-loop recyclability, supporting sustainable growth in photothermal coatings, while multi-walled carbon nanotubes (MWCNTs) improve self-healing (80 °C, 10 h) through photothermal effects and bond exchangeability in the epoxy vitrimer.^226^ In another study, Bohra *et al.* used graphene oxide and functional graphene oxide to create a self-healable epoxy vitrimer nanocomposite. In this study, graphene oxide (GO) was covalently functionalized with 4-AFD by reacting with thionyl chloride-modified GO and the amine groups of 4-AFD. The unmodified GO and functionalized GO (FGO) were mixed with a solution to create vitrimer epoxy (V-epoxy) composites. The thermal and mechanical characteristics of the composites were significantly improved by FGO, which qualified them for use in self-healing and shape memory applications. V-epoxy–FGO composites (0.5% and 1.0%) healed at 110 °C, whereas V-epoxy–GO composites (0.5% and 1.0%) healed at 90 °C due to the disulfide bond exchange reaction.^47^

## 5. Recycling of vitrimer nanocomposites: advanced techniques and performance enhancement

Recycling of vitrimers has gained significant attention due to their unique dynamic covalent bonds, allowing materials to exhibit excellent recyclability, stability, and performance after repeated processing.^54^ This review aims to discuss various recycling methods for vitrimer nanocomposites, highlighting their process efficiency, mechanical stability, and environmental implications.

### 5.1 Thermal processing: recyclability in vitrimers with heat activation

The primary advantage of vitrimers is that they can be recycled and reprocessed due to their dynamic covalent bond exchange processes. Unlike regular thermosets that cure irreversibly and cannot be reshaped, vitrimers can be moulded, welded, or repaired without compromising network integrity because they soften when heated above the topological freezing transition temperature (*T*_v_). Thermally driven dynamic bond exchanges that preserve the crosslink density while allowing chain mobility can be used to recycle this material. Leibler *et al.* were the first to show that transesterification reactions in epoxy vitrimers may be conducted repeatedly without compromising their mechanical qualities.^127^ According to more recent research, the type and concentration of the catalyst, the network architecture, and the interactions between the filler and the matrix all have a major impact on how effective thermal recyclability is. For example, the heat conductivity of epoxy vitrimers is enhanced by the addition of silica or carbon-based nanofillers. This speeds up stress release and increases the efficiency of reprocessing by facilitating heat dispersion during processing. However, some studies point out that too much filler may cause flaws or phase separation, which lowers the efficiency of thermal recycling.^72,161^ These findings demonstrate that one of the finest qualities of vitrimers is their heat-activated recyclability, but this property is highly dependent on their manufacturing process. Additionally, processing parameters like temperature, time, and pressure need to be appropriately regulated to avoid damage or insufficient bond exchange. All things considered, the combination of nanofiller reinforcement with heat-responsive vitrimer chemistry creates new opportunities for the development of high-performance, reprocessable materials. However, maintaining recyclability over time necessitates a careful balancing act between physical structure and dynamic chemistry.

**Fig. 10:**
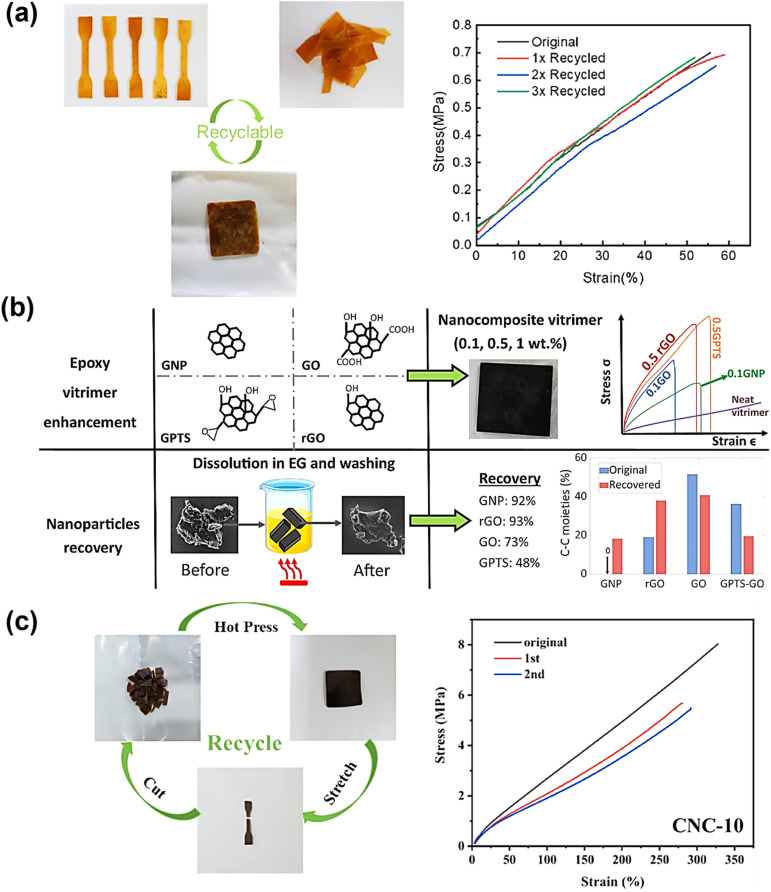
(a) Recycling and stress–strain curve of the original and recycled amine-modified PDMS and CNC vitrimer nanocomposite. Reproduced with permission from ref. 164 Copyright 2022 Elsevier Ltd. (b) Different nanofillers affect the enhancement of mechanical properties of the epoxy-based vitrimer nanocomposite and nanofiller recovery using EG solvent. Reproduced with permission from ref. 160 Copyright 2022 Elsevier Ltd. (c) Hot pressing of the ESO-cured XNBR/CNC vitrimer nanocomposite for recycling and stress–strain curve for the original and recycled nanocomposite. Reproduced with permission from ref. 230 Copyright 2022 Elsevier Ltd.

Sriharshitha *et al.*^227^ investigated the reshaping and self-healing abilities of a bio-silica reinforced polybenzoxazine vitrimer nanocomposite at ambient temperature, where damaged samples restored their original shape within 10 hours due to dynamic S–S bonds and hydrogen bonding interactions. Fourier-transform infrared (FTIR) spectroscopy verified the bond exchange processes, demonstrating effective recyclability without the need for external heat. Similarly, Wang *et al.*^164^ showcased the effectiveness of hot-pressing at 180 °C and 15 MPa for 1 h, which activated the amine-modified PDMS and CNC vitrimer nanocomposite network to exchange vinylogous urethane bonds dynamically. The nanocomposites exhibited 96% elongation recovery after thermal reshaping and maintained strong mechanical properties with no interface failures under tensile stress (Fig. 10a). Hajiali *et al.*^210^ have developed bio-based polymethyl methacrylate vitrimers and vitrimer nanocomposites reinforced by amine-functionalized polyhedral oligomeric silsesquioxane (POSS-NH_2_) nanoparticles to explore the influence of nanofiller amount in recyclability. The work focuses on reprocessing vitrimers and nanocomposite materials at a lower recycling temperature (125 °C) to reduce side reactions produced by active chain ends. Thermogravimetric analysis (TGA) was used to test the thermal stability of cured samples during recycling. The pure vitrimers and nanocomposite were mechanically reprocessed by grinding and hot-pressing at 125 °C for 14 metric tonnes. Specimens were recycled three times, and the network integrity was assessed using dynamic mechanical analysis (DMA), Fourier-transform infrared spectroscopy (FTIR), and tensile testing. The storage modulus and *T*_g_ of the recycled specimens were greater at room temperature than those of the pristine vitrimers. The initial reprocessed samples had a higher rubbery plateau modulus than the original samples, which might be attributed to the creation of more crosslinks within the system. The average recovery of mechanical characteristics for the nanocomposite was 88%. FTIR indicated no appreciable deterioration after several recycling cycles, and the peaks corresponding to vinylogous urethanes remained after recycling. Another study by Chen *et al.*^205^ emphasized the recyclability of hGnP/epoxy composites through powder grinding followed by hot pressing. Tensile tests indicated minimal strength reduction (6.2 MPa compared to 6.9 MPa for the original material), although elongation decreased due to aging and irreversible side reactions. These results highlight the feasibility of thermal recycling with negligible performance losses.

### 5.2 Solvent-based processing: recyclability in vitrimers with chemical reaction

Chemical recycling using solvent-mediated vitrimerization provides an effective approach for closed-loop and separate recycling of vitrimer matrices and fillers.^214^ Zhang *et al.*^84^ explored the reversibility of imine bonds in MWCNT-reinforced polyimine-based nanocomposites, employing heat-driven covalent bond exchanges facilitated by solvents. This process successfully achieved complete fracture healing, restoring tensile strength to 84–100%. However, a slight decline in conductivity over multiple cycles indicated a potential limitation for applications in electronics. Similarly, Poutrel *et al.*^160^ utilized solvents such as ethylene glycol (EG) to cleave dynamic bonds in epoxy vitrimer matrices, enabling the recovery of embedded carbon-based fillers. This technique preserved the physical properties of the fillers while maintaining the mechanical integrity of the recycled composites, demonstrating their efficacy in material separation and reuse (Fig. 10b). In a study, Zhou *et al.* showed that the conductive nanofillers could be readily recovered in a solution of DTT and DMF following filtering, much like carbon fiber composites, suggesting that this might be used for all kinds of reinforcements in vitrimer composite recycling pathways.^228^

### 5.3 Comparative analysis of virgin *versus* recycled vitrimer nanocomposites

A thorough comparison of virgin and recycled vitrimer nanocomposites underscores their ability to retain structural and mechanical properties even after repeated recycling cycles.^229^ Krishnakumar *et al.*^83^ investigated epoxy composites with GO fillers, observing that the flexural modulus recovered up to 88% after the first recycling cycle, although subsequent cycles showed reduced returns. The presence of dynamic disulfide bonds facilitated effective self-healing at 60 °C, but extended recycling cycles highlighted limitations in crosslink density and chain entanglement. Gao *et al.*^230^ demonstrated the robustness of ESO-cured XNBR/CNC composites, where cyclic reprocessing at 180 °C preserved mechanical properties comparable to those of the original material (Fig. 10c). This approach ensured consistent stress–strain behavior across cycles, affirming the effectiveness of dynamic covalent bonds in maintaining material integrity.

### 5.4 Energy efficiency and environmental impact of vitrimer nanocomposites

Recycling methods such as hot-pressing and solvent-based vitrimerization play a crucial role in waste reduction and sustainability. Hot-pressing eliminates the need for additional chemicals, thereby minimizing environmental impact, while solvent-based techniques enable the recovery of high-value fillers like carbon fibers without compromising their properties. Yue *et al.*^167^ emphasized the necessity of refining recycling processes to uphold thermal stability and mechanical performance. Their research demonstrated that vitrimerized epoxy/CNC composites could be reshaped and recycled effectively, retaining thermal properties and exhibiting shape memory capabilities.

### 5.5 Longevity and stability of vitrimers over multiple recycling cycles

Ensuring the stability of vitrimers across multiple recycling cycles is essential for their practical use. Ran *et al.*^231^ found that increasing CNF loading in vitrimer composites extended stress relaxation times while enabling thermal recycling with consistent stability. This highlights the importance of balancing filler content with recyclability efficiency. Liu *et al.*^232^ demonstrated the rapid self-healing ability of EVA–GOB composites through NIR-triggered bond exchange reactions. After five hours of healing, the composite's mechanical properties, including tensile strength and toughness, were nearly identical to those of the original sample, confirming the durability of recycled materials.

Recycling vitrimer nanocomposites using thermal and solvent-based approaches effectively preserves their mechanical and structural properties while minimizing environmental impact. Dynamic covalent chemistry facilitates efficient reshaping, self-healing, and material recovery, establishing vitrimers as a sustainable solution for future applications. Further advancements in recycling techniques could improve the durability and performance of these materials across various industries.

A comparison of different recycling techniques for vitrimer-based nanocomposites is provided in Table 2, which also highlights important performance attributes such as self-healing properties, mechanical retention, and process efficiency. Interestingly, a number of studies demonstrate how dynamic covalent chemistry, including disulfide and imine bond swaps, might be advantageous in enabling robust mechanical recovery and recyclability.

**Table 2:** Summary of Recycling Techniques and Performance

S. no.	Recycling technique	Process efficiency	Stability	Mechanical retention	Self-healing	Key observations	Ref.
1	Room temperature reshaping	High: bond deformation & reformation evident	Stable due to dynamic S–S and H-bonding	Satisfactory across various weight ratios	Effective reshaping within 10 hours	FTIR confirms bond exchange mechanisms	227
2	Thermal hot pressing (180 °C)	High: efficient surface smoothness retained	Stable; 96% elongation recovery	Maintained good tensile properties	Interfaces bonded well chemically	No interface failure during tensile testing	164
3	Thermal recycling	—	—	—	—	—	161
4	Heat-driven imine bond exchange	High: 84–100% tensile strength recovery	Slight conductivity degradation	Recoverable tensile strength	Healed cracks invisible to the naked eye	Repeated healing shows minor conductivity loss	84
5	Powder grinding & hot pressing	High: minimal performance loss	Stable; aging-induced elongation reduction	Nearly identical tensile strength	Not explicitly reported	Recycled composites showed negligible strength reduction	205
6	Low-temp (60 °C) healing & hot pressing	Moderate: dependent on GO content	Decreasing modulus with repeated healing	73–88% recovery after two cycles	Facilitated by disulfide bond exchanges	Recycled composites retain efficient self-healing properties	83
7	Topological rearrangement (180 °C)	High: cyclic reprocessing yields consistent	Stable across cycles; smooth surface	Similar stress–strain curves over cycles	Not reported	Favorable reprocessing using β-hydroxy ester bonds	230
8	Grinding & hot pressing above *T*_g_	Moderate: effective for reshaping	Stable due to chemical bonding with CNCs	Good thermal stability post-recycling	Not reported	Efficient shape memory applications	167

For example, Sriharshitha *et al.*^227^ prepared an environmentally friendly bio-based polybenzoxazine vitrimer that contains thiol groups (SH) and bio-silica (BS) and has the capacity to self-heal or recycle. This study demonstrated high efficiency in room-temperature reshaping made possible by S–S and H-bond interactions, with FTIR analysis confirming good mechanical retention and reshaping capabilities. In a similar vein, Wang *et al.*^164^ used thermal hot pressing for a polydimethylsiloxane composite reinforced with a cellulose nanocrystal to obtain high surface integrity and 96% elongation recovery, with perfect interface bonding that increases endurance. The remarkable tensile strength recovery (84–100%) and almost undetectable repaired cracks of Zhang *et al.*'s imine-based vitrimer system reinforced with MWCNT suggest the potential of dynamic imine chemistry for repetitive healing applications.^84^ The resilience of β-hydroxy ester and powder-pressed systems was emphasised by Gao *et al.*^230^ and Chen *et al.*,^205^ who discovered no performance loss throughout recycling cycles. The other entrants, including Krishnakumar *et al.*^83^ and Yue *et al.*,^167^ demonstrated how low-temperature and CNC-assisted systems, respectively, contribute to recyclability and shape memory behaviour. Nevertheless, after numerous cycles, a number of approaches showed reduced modulus or moderate conductivity loss, suggesting room for long-term performance optimisation. All of these results highlight the value of vitrimer chemistry in facilitating energy-efficient, repeatable recycling processes. Because of their exceptional mechanical properties and, in certain cases, their capacity for self-healing, vitrimers are promising options for circular, sustainable material systems.

## 6. Applications of nanofiller-enhanced vitrimers

In this section, many possible applications of vitrimers illustrating their potential for innovation and influence are discussed. Vitrimers possess the capability to replace conventional thermosetting polymers through diverse applications owing to their distinctive amalgamation of chemical and physical characteristics, particularly at operational temperatures. Vitrimers possess many exciting properties, including weldability, self-healability, reconfiguration, recyclability, and malleability, rendering them particularly appealing for sustainable applications in various industries. Their capacity for reversible chemical cross-linking and facilitating modifications in the networks of polymers are the reasons for their sustained excellent performance even at increasing temperatures. Moreover, the adaptability of vitrimers, propelled by progress in material design, unveils a wide array of novel applications and opportunities.

### 6.1 Vitrimer nanocomposites in automotive and aerospace components

This section explores the recent advancements in potential applications of nanofiller-enhanced vitrimers. Sabet, in his study, showed that self-healing graphene polymer nanocomposites can be revolutionary in the aviation industry. Such materials are lightweight in construction, possess excellent mechanical properties, and damage can be autonomously self-healed, which are critical for aviation. The amalgamation of such biomaterials with smart agents allows rapid healing of damage like cracks or delamination on the aircraft's fuselage and wing components. A breakthrough highlighted is the use of microvascular networks to transport healing agents to the site of damage, simulating biological systems. It ensures that this specific repairing approach improves the structural and operational reliability of aircraft. In addition, these nanocomposites also play an important role in weight reduction, thus improving fuel consumption and increasing payloads. New fabrication techniques like additive manufacturing not only make lightweight components more complex but can also embed the structural component with self-healing properties.^233^ In this interesting study, Pandey *et al.* found self-healing polymers as one of the breakthroughs in safety and sustainability in aviation. In particular, self-healing polymers, especially those with intrinsic and extrinsic mechanisms, can withstand some critical problems of aviation components, including catastrophic failure and limited service life. These materials have an advantage, particularly in extreme environments, that will be experienced by the aircraft in terms of high temperature, pressure, and aerodynamic loads.^234^ Yang *et al.* described the usage of epoxy vitrimers in aerospace because they combine thermoset mechanical and thermal stability with thermoplastic recyclability and reprocessability. These materials surpass limits such as maximum working temperature and high mechanical stresses, hence increasing the service life of components and assemblies. Functional components such as graphene/carbon nanotubes are used to improve mechanical strength, thermal management, and environmental response. The combined features of recyclability and reprocessability help to achieve eco-friendly aims by minimizing waste and allowing the recovery of costly components such as carbon fiber reinforcements. This transition in epoxy vitrimers aims to improve performance and environmental friendliness for the next generation.^22^ Kausar *et al.* showed how to design and implement self-healing polymer-based nanocomposites for aerospace engineering. These inclusions are designed to integrate nanoparticles such as carbon nanotubes, graphene, and functionalized nanocapsules to enable damage repair mechanisms such as interdiffusion of polymer chains or reversible chemical bonding. Among key applications in the aerospace sector are functional components, such as fuselage panels, surface coatings, and engine parts.^235^ Another review by Kausar *et al.* considers incorporating carbonaceous fillers like graphene and carbon nanotubes in shape-memory polymers for aerospace purposes. Such advanced materials possess remarkable stimuli-responsive capabilities to recover structural damage in spacecraft and aircraft parts. The use of effective dispersion and alignment of the nanofillers and crosslinking of polymer chains increased performance. Applications include shock-resistant fuselages, morphing wings, antennas, and engine parts. The materials have benefits such as low weight, high thermal and mechanical strength, and anti-corrosion properties. On the downside, there are issues with nanoparticle agglomeration and the cost of processing. The article notes the need to adopt advanced techniques, such as 4D printing, to overcome these issues while improving the strength and functions of aerospace structures.^236^

### 6.2 Vitrimer nanocomposites in electronics and conductive materials

**Fig. 11:**
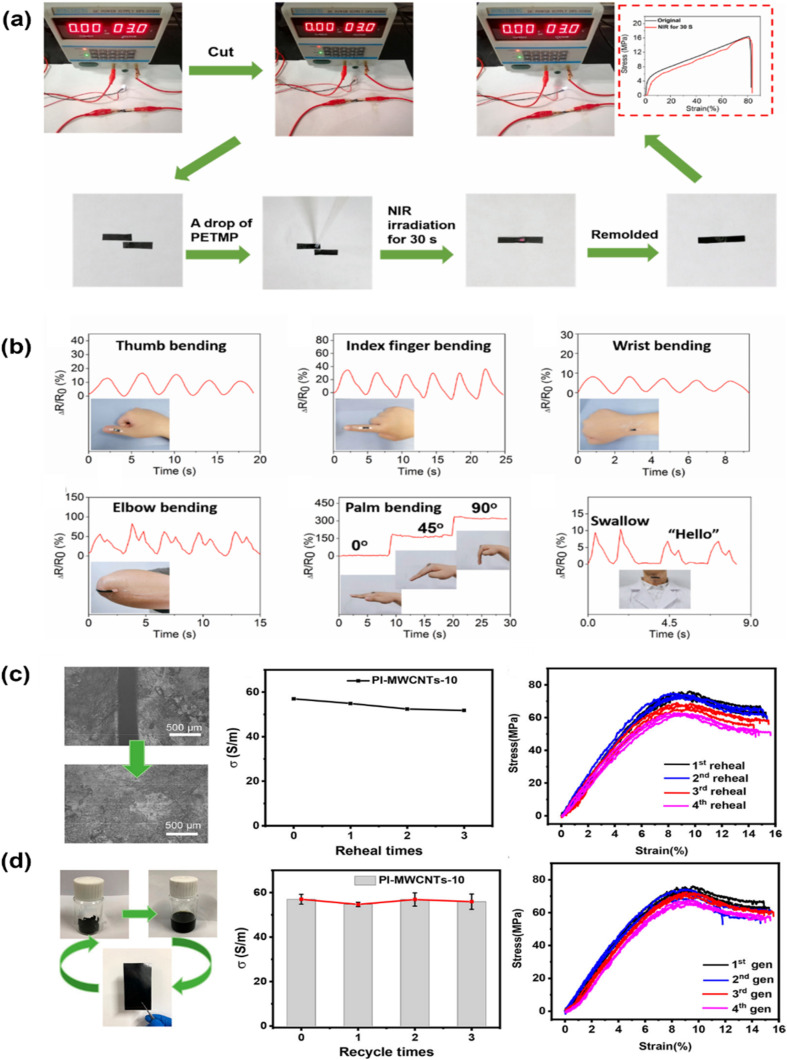
(a) Real-life images of the photo-welding method of the EPCNT_5_ vitrimer nanocomposite. (b) Detection of various human motions by using the EPCNT_5_-skin sensor. Reproduced with permission from ref. 237 Copyright 2022 Elsevier Ltd. (c) Optical microscope images of a rehealed PI-MWCNT-10 film along with their conductivity and mechanical properties. (d) Recycling process of a PI-MWCNT-10 film with the conductivity and mechanical properties of the original and recycled nanocomposites. Reproduced with permission from ref. 84 Copyright 2020 American Chemical Society.

In recent years, electronic and conductive materials have garnered significant attention in research, with vitrimers playing a crucial role in this area. Therefore, this section discusses the applications of vitrimers in the electronics field. The research by Guo *et al.* presents the idea of creating a series of carbon nanotube-embedded vitrimer nanocomposites (EPCNT_*x*_), where the integration of electrical conductivity and other unique characteristics such as the ability to be reprocessed, biodegraded, and photo-welded are aimed at. EPCNT_5_, which had 5 wt% multi-walled carbon nanotubes (MWCNTs), was a notable CNT–epoxy. It was claimed that this composite had the highest electrical conductivity of 1.63 S m^−1^, surpassing many CNT–epoxy composites presented in previous studies. The epoxy-based composites also exhibited a uniform dispersion of MWCNTs, which was believed to have minimized its agglomeration, thus mass-improving its electrical and mechanical behaviors. Considering these results, EPCNT_5_ composites were also proposed to have good mechanical properties, such as tensile stress and fracture strain, which stood at an average of 16.3 MPa and 81.8%, respectively. The composites were thermally characterized by analyzing the glass transition temperature (*T*_g_) using differential scanning calorimetry. This resulted in a *T*_g_ of 47.9 °C for EPCNT_5_, which is useful for skin outputs but not exclusively restricted to that area. Stress relaxation tests were performed to observe the multifunctional, dynamic, and reprocessable nature of the bonds formed in the covalent network, suggesting that the composites retained strength. Greater than 140 °C temperatures were recorded for EPCNT_5_ composites when exposed to near-infrared light, providing the EPCNT_5_ with excellent photothermal properties, resulting in quicker photo-welding processes (Fig. 11a). The bond networks allowed the repairing of material fractures without any electrical damage and in a quick and pressure-free manner. Further investigation was conducted on the effect and use of EPCNT_5_ composites in skin sensing. The need for movement of human joints and throat vibration sensing was extenuated by the stability of the EPCNT_5_ composites, indicating its adaptation for use in various electronic goods worn on the body (Fig. 11b). The dynamic covalent bond-based vitrimer matrix facilitates material decomposition and MWCNT retrieval, which solves the environmental problems associated with conventional thermosetting plastics. This new material opens up a horizon of possibilities for applications in flexible electronics.^237^ Zhang *et al.* describe a new version of polyimine vitrimers, composites of multi-walled carbon nanotubes (MWCNTs) for materials from the wide field of flexible electronics. MWCNTs exist in low proportions and yield significantly lower than 0.05 S per m bulk composites. An appropriate composite, PI-MWCNT-10, reached a maximum electrical conductivity of 57 S m^−1^ when 10 wt% MWCNTs was mixed in. Additionally, the composite fared well in mechanical aspects, attaining a tensile strength of 74 MPa and Young's modulus of 1.2 GPa, which is improved compared to pure polyimine matrices, which only enjoyed 3.65 S m^−1^. Furthermore, the polyimine 10 composite also enjoyed good ductility, boasting the ability to be reshaped into different and various configurations due to the fact that the electrical aspect of the materials remained intact throughout the process. The results also indicated that less than four percent of the strength of the composite was lost when it was repeatedly reshaped and stretched several times. The dynamic imine bond exchange in the nanocomposite made it self-healable and reprocessable. As shown in Fig. 11c and d, the electrical conductivity of the bond was restored to 97% through 3 healing cycles, and it was depolymerized in an amine solution, showing the ease with which MWCNTs can be recovered, allowing for more polyimine components to be retrieved and utilized later. The material's endurance was impressive when articles made of recycled composites were put through repeatable testing. It showed almost 100 percent permeability to MWCNT C2 and the conductivity components even after extensive tensile strain of over 90 percent. The outcomes indicate a promising set of materials that reduces waste and helps the ecosystem by offering a ‘green’ and sustainable precursor for flexible electronic materials. The future's flexible sensors and smart devices can be durable, low-cost, and eco-friendly, as the research highlights the opportunities for using MWCNT–vitrimer composites in electronics.^84^ Luo *et al.* developed a vitrimer–graphene aerogel (GA) nanocomposite that has excellent conductivity and flexibility due to its composition of a polyimine matrix and a 3D-connected GA network. The electrical conductivity of the composite at 5% GA concentration was 161 S m^−1^, which was much greater than that of multi-walled carbon nanotubes (5 S m^−1^) or commercial graphene powder (19 S m^−1^). The conductivity was nearly 2700 times larger than that of conventional counterparts, reaching 135 S m^−1^ even at a 3 wt% GA content. The composite is a promising solution for environmentally friendly flexible electronics since it can retain its shape and form while maintaining conductivity.^238^ Lorero *et al.* explored the use of vitrimer nanocomposites based on carbon nanotubes (CNT) in electronics. The crosslinked epoxy vitrimer matrix with dynamic disulfide bonds was found to be economically beneficial. The materials showed electrical conductivity, facilitating information exchange and allowing Joule heating with low voltage. These properties enable easy reprocessing and low-heat welding of parts. Key mechanical properties were retained at a glass transition temperature of 170 °C, and tensile strength losses were limited to 77% after heating CNTs. The study highlights the potential of vitrimer nanocomposites in future electrical construction and structure-forming elements, demonstrating their potential in flexible electronic devices and materials that consume minimal energy.^49^ Gómez-Sánchez *et al.* developed carbon nanotube-reinforced self-healing vitrimers using epoxy, which have potential for Self-Healing Mechanism (SHM) applications. The materials are dynamically active, allowing self-healing and strain measurements. The study revealed that the increase in the CNT content significantly improved the conductivity of the materials, reaching 0.30 S m^−1^ at the CNT loading of 0.2 wt%. The addition of AFD with 10% excess over stoichiometry enhanced tensile strength by 45%, reaching 80.18 MPa from the stoichiometric formulations. However, anything above this led to a reduction in the tensile strength, accounting for a decrease in the crosslink density by 41%. Strain sensitivity evaluated through the gauge factor, GF, was also promising, with low and high values of 0.69 and 2.22, respectively, depending on the concentrations of CNT and 2-aminophenyl disulfide (AFD). The materials show excellent real-time damage and strain monitoring, making them suitable for integration into structural health monitoring (SHM) systems. Self-repairing vitrimer composites could be used in aerospace and wind energy structures, where real-time damage monitoring and intelligent functionality can improve safety and lifespan.^239^

### 6.3 Vitrimer nanocomposites in self-healing applications

The unique combination of dynamic covalent chemistry and structural reinforcement by nanofillers has led to growing interest in vitrimer nanocomposites for self-healing applications. Under the influence of heat, light, or other stimuli, these materials can self-heal microcracks and mechanical damage without sacrificing the integrity of the cross-linked network, due to associative bond exchange mechanisms like transesterification, urethane–hydroxyl exchange, urethane–urethane exchange, disulfide exchange, and imine metathesis.^133,240–243^ The self-healing efficiency is further increased by adding nanofillers like graphene oxide (GO), cellulose nanocrystals (CNCs), silica nanoparticles, or carbon nanotubes (CNTs), which improve filler–matrix interaction, stress transmission, and thermal conductivity.^244^ For example, enhanced interfacial energy dissipation and localised heating have demonstrated faster mechanical recovery and healing rates in GO-reinforced epoxy vitrimers.^232,245^ Light-induced self-healing is also made possible by the use of photothermal fillers, such as carbon nanotubes (CNTs), which enable accurate, spatially controlled repair without the need for external heating.^246^ Bio-based nanofillers, like CNCs, are also suitable for sustainable material design because they provide flexibility and reactivity while maintaining recyclability. In coatings, electronics, automotive components, and smart textiles, where material longevity, repeatability, and damage tolerance are critical, these self-healing vitrimer nanocomposites offer exciting potential uses. In general, the integration of vitrimer chemistry and nanofiller activity offers a flexible framework for creating materials that are flexible, robust, and reprocessable for practical self-healing applications.

**Fig. 12:**
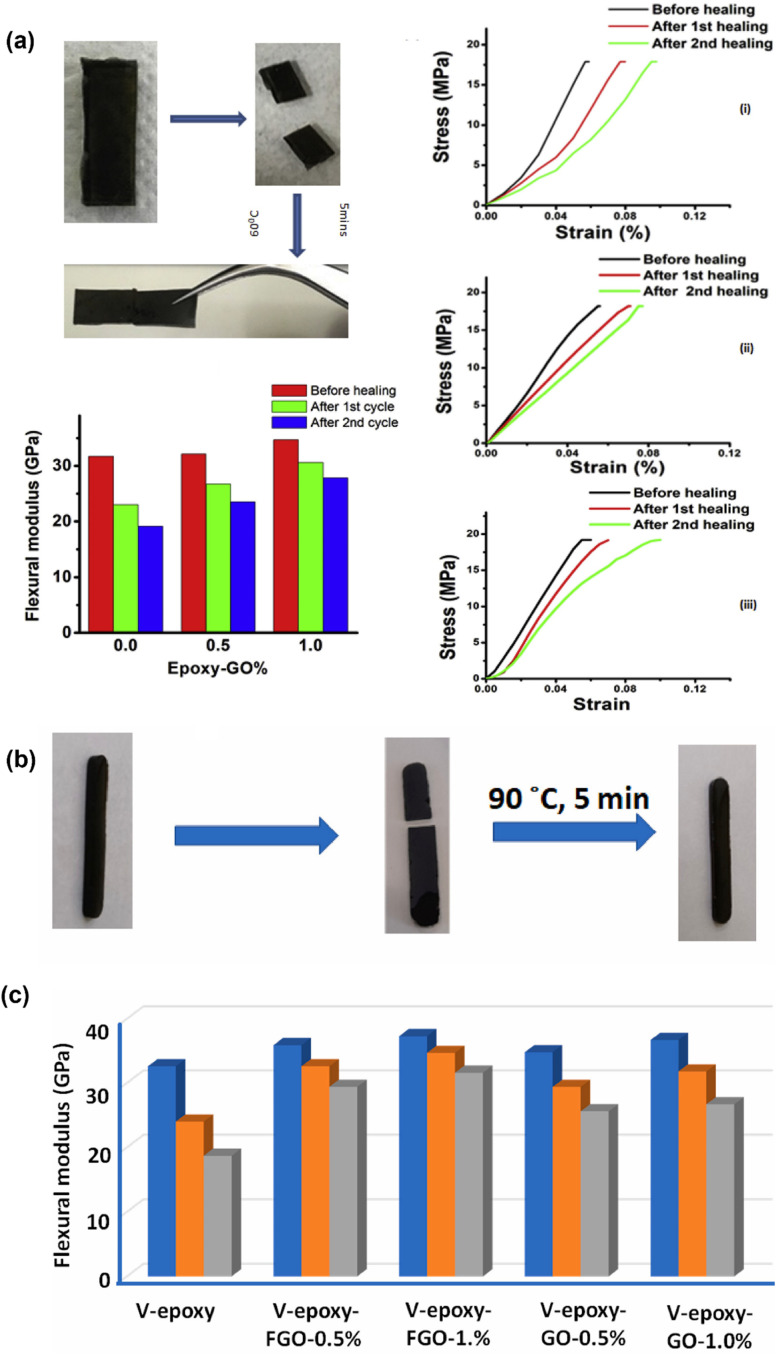
(a) Real-life images of uncut, cut, and healed EP-1% samples along with stress–strain curves for representing the healing behavior of (i) EP–pristine, (ii) EP-0.5%, and (iii) EP-1% specimens and changes in flexural modulus after various healing cycles for different samples. Reproduced with permission from ref. 83 Copyright 2019 Elsevier Ltd. (b) Real-life images of uncut, cut, and healed specimens of V epoxy-GO-1%. (c) Variation of flexural modulus with respect to different healing cycles for the different specimens (blue bar-pristine sample; orange bar-first healing cycle; gray bar-second healing cycle). Reproduced with permission from ref. 47 Copyright 2023 Elsevier Ltd.

The study by Krishnakumar *et al.* considers the synthesis of epoxy vitrimer nanocomposites reinforced with graphene oxide (GO) so that the self-healing characteristics of the material are developed *via* a disulfide exchange-based covalent adaptive network. The self-healing behavior of these nanocomposites is catalysis-free, and shape memory and mechanical properties are enhanced due to the addition of GO.^83^ As shown in Fig. 12a, the inclusion of 1 wt% GO causes a drastic improvement in self-healing efficiency to 88% and 80% after the first and second cycles of healing, respectively, at 80 °C. So, 1 wt% GO lowered the glass transition temperature, *T*_g_, to 53 °C, making low-temperature self-healing possible. There were 7.1% and 9.4% higher flexural strength and flexural modulus for the materials composed of GO with vitrimers than those composed of only vitrimers. Stress relaxation experiments confirmed a fast disulfide bond exchange mechanism owing to relaxation times of 112.8 s at 60 °C and 34 s at 80 °C. It is also illustrated that 73–88% of the material's flexural modulus is recoverable, suggesting high durability and the capability to reuse it. The self-healing ability of the nanocomposites enabled them to recover their original shape with 100% fullness while retaining 1 wt% GO at 80 °C. This combination of strengths or key mechanical and thermal properties makes these materials prime for structural repair and self-healing applications.^83^ In another study, Bohra *et al.* focused on optimizing the self-healing properties in vitrimer nanocomposites using functionalized graphene oxide (FGO). The researchers modified graphene oxide to improve wettability and reduce agglomeration, enhancing thermal, mechanical, and self-healing characteristics. The efficiency of self-healing was examined with different filler concentrations, where V-epoxy–FGO-0.5% and V-epoxy–FGO-1.0% reached healing temperatures of 110 °C after five minutes compared to 90 °C in their GO counterparts (Fig. 12b). After being healed, flexural modulus values demonstrated a high degree of retention, and after several cycles, further increases were seen due to the modulus-increasing viscoelasticity. For example, V-epoxy–FGO-1.0% exhibited 34.7 GPa of flexural modulus once the first healing cycle was done and 31.6 GPa once the second cycle was finished (Fig. 12c). Shape memory testing showed that FGO-based composites had a high % recovery rate of 98% compared to 91–93% for both GO and pure vitrimer composites. In terms of thermal analyses, a 10 °C increase in glass transition temperature (*T*_g_) (which ranges from 95–105 °C) and improvement in decomposition temperature were noted, where the weight loss temperatures of 5% and 50% improved by 15 °C and 7 °C respectively, in the case of 2% FGO modification. The results highlight the efficacy of FGO-based vitrimer nanocomposites for self-healing purposes with improved mechanical and thermal properties.^47^ Park *et al.* paid attention to the enhanced self-healing characteristics of graphene oxide-reinforced vitrimer nanocomposites by molecular dynamics simulation. The analysis demonstrated that the addition of graphene oxide lowers the glass transition temperature of the vitrimer, making the self-healing procedure of the composite possible at reduced temperatures. Concerning the self-healing properties, the GO/vitrimer nanocomposites were superior to even the unstretched vitrimer under every test that was performed. Almost all self-healing was achieved in the GO/vitrimer at 400 K, and the polymer remained crosslinked when being stretched, while some debonding was seen in the unstressed vitrimer. GO/vitrimer could self-heal completely at 370 K, above its *T*_g_ but lower than that of the unstressed all-crosslinked polymers. In contrast, the unstressed all-polymer failed to self-heal. Even at 300 K, lower than *T*_g_, the GO/vitrimer self-healed better than the unstressed all-polymer. This is believed to be because of the increased exchange of bonds that resulted from enhanced molecular movement. Additionally, over 90% of the newly formed disulfide bonds broke when the simulation temperature increased, which aided in breaking bonds and self-healing. The results highlighted the efficacy of the GO-reinforced vitrimer nanocomposites as self-healing systems applicable for several other purposes, as the molecular scale analysis showed improvement in healing action.^74^

### 6.4 Vitrimer nanocomposites in adhesives and coatings

Because of their exceptional ability to combine dynamic covalent bond exchange with permanent cross-linked network integrity, vitrimer nanocomposites have emerged as feasible options for next-generation adhesives and coatings.^219,247–250^ This allows for reworkability, repairability, and recyclability, which are typically absent in normal thermoset-based formulations. In addition to increasing the matrix's mechanical strength and barrier properties, nanofillers such as silica nanoparticles, graphene, carbon nanotubes (CNTs), and clay improve chemical resistance, thermal stability, and substrate adhesion, all of which are important for adhesive and coating applications. When surface-functionalized, these nanofillers can enhance interfacial bonding, provide electrical or thermal conductivity, and enable stimuli-responsive behaviour. For example, under thermal or photothermal stimuli, vitrimer adhesives containing CNTs or graphene exhibit improved weldability and debonding control, allowing for precise component assembly and disassembly in high-performance systems.^226,251–253^ The scratch resistance, self-healing, and anti-corrosion properties of vitrimer nanocomposites in coatings make them suitable for protective and functional surface layers in electronics, automotive, aerospace, and marine applications. End-of-life recycling and sustainable manufacturing are also enabled by their ability to undergo heat reprocessing and reshaping. Together, vitrimer chemistry and nanoscale reinforcement provide a formidable arsenal for developing intelligent, long-lasting, and environmentally friendly adhesives and coatings with cutting-edge qualities suitable for challenging conditions.

**Fig. 13:**
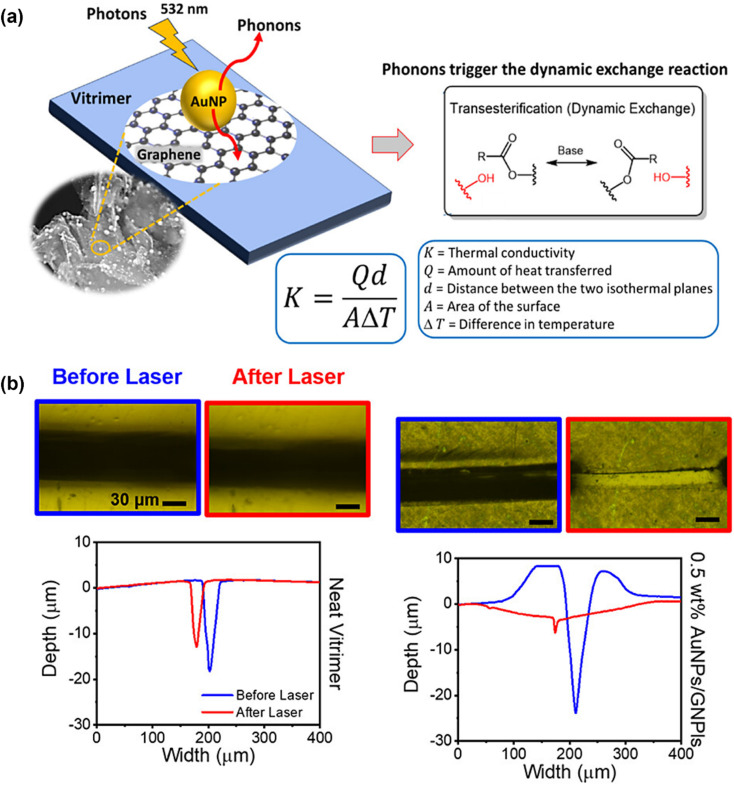
(a) A schematic illustration of photothermal conversion of AuNPs coated on a graphene nanoplatelet on top of a vitrimer matrix. (b) Images of the damaged surface after the laser exposure neat vitrimer (left) and 0.5 wt% AuNPs/GNPIs (right). Reproduced with permission from ref. 86 Copyright 2024 American Chemical Society.

The article by Ren *et al.* presents the concept of creating self-repairing nanocomposite coatings that provide tightly held and coating features. In this composite, gold nanoparticles (AuNPs) and graphene nanoplatelets (GNPIs) are incorporated into a vitrimetric matrix with the intention of utilizing their photothermal properties for ultrafast self-healing capability. The composites absorb 532 nm laser light, and as these are photothermal materials, they undergo LSPR (Localized Surface Plasmon Resonance) followed by photothermal conversion, thus augmenting plasmonic heating (Fig. 13a). The results showed that adding 0.5 wt% AuNPs/GNPIs elevated the recovery rate of surface scratches from 11.5% for neat vitrimer films to 90.9% within 100 milliseconds (Fig. 13b). Profilometry and microscopy showed that the damaged areas were closed within seconds. The composites also improved thermal properties, as *T*_5%_ increased to 355 °C and *T*_g_ increased from 32 °C to 35 °C. Molecular dynamics modeling captured the local temperatures of the fillers to be above 350 °C, demonstrating dynamic covalent bond exchange for self-healing. These features suggest the vitrimer nanocomposites are suitable for industrial adhesives and coatings due to their rapid mechanical flexibility and reconfigurability, enhancing repair turnaround and material durability. This work reinforces the efficiency of light-activated coatings as important elements for future engineering endeavors.^86^ Legrand *et al.* developed epoxy-based vitrimer nanocomposites filled with silica nanoparticles for adhesives and coatings. These nanocomposites were produced using a solvent-free method, with filler loadings of up to 40 wt%. The addition of silica nanoparticles enhanced the modulus, thermal, and mechanical properties and did not affect the topological rearrangement of the vitrimer. Modified silica nanoparticles improved interfacial bonding and dispersion of fillers, reducing stress relaxation time by 25% compared to non-functionalized composites. Mechanical tensile strength tests showed a 14 MPa increase for functionalized silica composites. These materials have good weldability, reshaping properties, and shear strengths, making them suitable for industrial adhesives and coating applications with long lifetimes and ease of recycling and processing.^72^ Lorwanishpaisarn *et al.* focused on utilizing vitrimer nanocomposites as coatings that offer self-healing capabilities. The composites were synthesized from bio-sourced resins containing cashew nutshell liquid (CNSL) or citric acid (CA) as well as multi-walled carbon nanotubes (CNTs). Such materials work through dynamic bond exchange reactions, which enable healing and restructuring with the support of low-level NIR light. The experimental evidence shows that introducing 0.5 wt% CNTs in a vitrimer–CNT (V–CNT0.5) nanocomposite considerably increases the performance of the coatings and anti-corrosion properties. The self-healing efficiency, which is assessed in terms of Shore D hardness, improved from 94.91% in the formulations without CNTs to 97.3% in those that utilized the CNTs. Further, the storage modulus increased from 5773 MPa (without CNTs) to 8602 MPa (with CNTs), and the glass transition temperature (*T*_g_) improved from 27.3 °C to 30.6 °C. Coated samples of steel with V–CNT0.5 also exhibited 99.99% anti-corrosion efficiency, as shown by the corrosion protection tests, and the corrosion rate improved from 9.53 × 10^2^ MPY to 3.12 × 10^−5^ MPY. These results confirm the ability of these nanocomposite forms to be used as industrial coatings that are environmentally friendly and efficient in strength, whereby the coating has self-healing capabilities alongside its corrosion resistance features. This invention solves the central problem of the ability of the structures to maintain stability over time, along with the high maintenance cost.^48^

### 6.5 Vitrimer nanocomposites in biomedical applications

Vitrimer nanocomposites are becoming more popular in the biomedical field because they are flexible, biocompatible, and can be recycled. These traits make them good for uses that need performance that can be changed, shape memory, and the ability to heal themselves.^254^ Biocompatible nanofillers like cellulose nanocrystals (CNCs), graphene oxide (GO), and silica nanoparticles are added to vitrimer matrices to make biomedical devices stronger, more active, and better at interacting with cells.^255–257^ The dynamic covalent exchange processes in these nanocomposites, like transesterification or disulfide exchange, let minimally invasive implants, wound dressings, and tissue scaffolds heal and reshape themselves with heat or light. Also, when making drug delivery systems and resorbable scaffolds for tissue engineering, vitrimers' ability to be injected, reused, and have adjustable degradation rates is helpful. Functional nanofillers can make these composites respond to stimuli, release drugs in a specific way, or have antibacterial properties, depending on how they are used. As the need for smart and sustainable biomaterials grows, vitrimer nanocomposites provide a good foundation for building next-generation medical devices and regenerative systems that are stronger, work better, and are better for the environment.

**Fig. 14:**
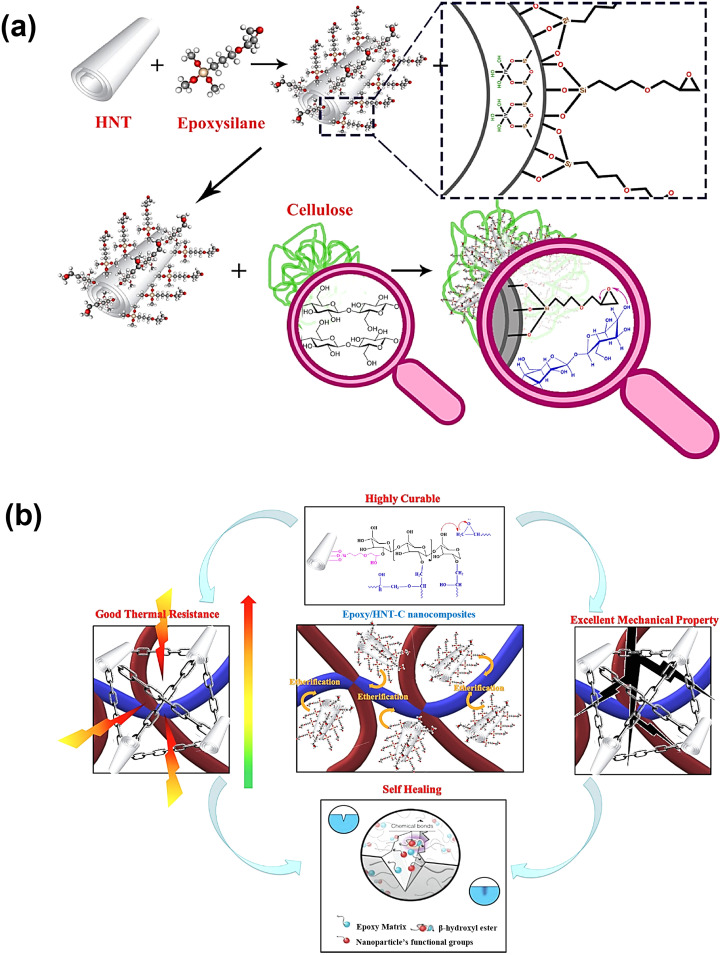
(a) Schematic representation of the surface modification method used in treating HNT with cellulose using EPPTMS as a coupling agent. (b) Schematic representation to correlate curability (structural property), thermal and mechanical (bulk properties), and self-healing (surface property) behaviors of the developed vitrimer nanocomposite coatings. Reproduced with permission from ref. 89 Copyright 2020 Elsevier B.V.

The article by Jouyandeh *et al.* aims to produce new types of coatings for biomedical applications characterized by self-healing and eco-friendly properties. The authors prepared cellulose-functionalized halloysite nanotubes (HNT-C) and added them to the epoxy resin to obtain polyfunctional vitrimer-like ones (Fig. 14a). The results showed that the addition of 0.3 wt% HNT-C could accelerate the curing process since the heat of cure (Δ*H*) value rose more than three times from 129 J g^−1^ for the neat epoxy to 456 J g^−1^ for the composite. HNT-C addition yielded a composite mechanical improvement with 16% in tensile strength and 56% in its elongation at break compared to those of the neat epoxy. The coatings showed remarkable thermal stability with decomposition temperatures (*T*_5%_) of neat epoxy starting at 173.10 °C and remaining at 179.14 °C even after adding the HNT-C nanocomposite. There was also an increase in glass transition temperature (*T*_g_) up to 142 °C, which suggests a high level of crosslinking with high stability. Self-healing is most likely ascribed to the transesterification reactions in the system, while cellulose functionalization facilitated the relaxation process. As shown in Fig. 14b, at higher temperatures, rapid self-healing was observed in the nanocomposites, demonstrating possibilities of use in biomedical devices where hard, self-healing, and biocompatible coatings are desired.^89^ The review by Kaur *et al.* provides an overview of the prospects of vitrimers for medical applications. It focuses on the vitrimers' application in tissue engineering, drug delivery systems, and biodegradable implants on account of their biological compatibility, self-healing, and recycling features. The dynamic covalent bond of the vitrimers enhances stress relaxation, which self-heals minor defects in biomedical devices. For instance, some network rearrangements will reduce the stress relaxation time, thus allowing these materials to be received and stored under mechanical stress repeatedly without damaging their structure. In the field of drug delivery systems, vitrimer-based systems can respond to triggers such as pH or temperature, thus controlling the release of drugs. The study points out the challenges that include controlling dynamic bond chemistry for consistency in a biological context and *vice versa*, such as concentration of salts. The discussion makes a strong case for vitrimers as a game-changing material that alters biomedical engineering and healthcare systems.^258^

## 7. Environmental and economic benefits of vitrimer nanocomposites

By virtue of economic and environmental compatibility, vitrimer nanocomposite development has become a major success in the search for sustainable materials. Vitrimers bridge the gap between thermoplastics and thermosets due to their ability to form dynamic covalent bonds that help in recycling and reprocessing with minimal deviation from the desirable properties of the material. This review has been focused on an expanded analysis of resource efficiency, life cycle analysis, carbon footprint reduction, and economic attainability of vitrimers as a modern-day material. Vitrimer nanocomposites substantially enhance resource efficiency by facilitating recycling of materials generally deemed waste in conventional thermosetting applications. The capacity to reprocess vitrimers enables producers to decrease raw material requirements, consequently alleviating the environmental impact linked to the extraction of materials and manufacturing. Employing renewable feedstocks like plant-based oils and lignin-derived chemicals for vitrimer production can reduce dependence on petrochemical resources. This transformation conserves limited resources and fosters a circular economy in which materials are reused instead of discarded after one use.^258,259^ A comprehensive life cycle analysis (LCA) comparing conventional recycling approaches with vitrimerization technology reveals that vitrimers often have a lower environmental effect.

Conventional thermoset recycling techniques generally require energy-intensive procedures that may produce considerable waste. Conversely, the dynamic characteristics of vitrimer networks facilitate simpler processing and reconfiguration at lower temperatures, perhaps resulting in less energy usage over their lifespan. Research demonstrates that the life cycle assessment of vitrimers indicates potential for reducing greenhouse gas emissions relative to traditional materials, underscoring their prospective contribution to sustainable manufacturing methods.^258,260^ The introduction of vitrimerization strategies into the manufacturing process mainly aims to reduce the carbon footprint. Due to their ability to self-heal and recycle, vitrimers increase their products' lifespan, reducing the frequency of replacements and diminishing the associated resource utilization. Production of bio-based vitrimers from diverse natural sources significantly decreases the carbon emissions associated with the alternative manufacture of synthetic polymers.^55^ From an economic standpoint, vitrimer nanocomposites are a cheaper solution than standard materials. Over time, the ability to recycle and reprocess these materials decreases manufacturing costs by cutting raw material prices and waste disposal fees. Additionally, due to their eco-friendly properties, materials manufactured from vitrimers are projected to acquire market momentum as corporations put a larger focus on sustainability. In conclusion, vitrimer nanocomposites have a significant potential to tackle the current environmental condition without hindering the production of materials or altering the quality of the materials.

## 8. Future prospects and innovations in vitrimer technology

Recent breakthroughs in vitrimer technology have focused on increasing their characteristics and broadening their usage *via* new chemistries. A key field of study is dynamic covalent networks, which describe the vitrimers owing to dynamic covalent bonds that allow for reversible reactions under specified conditions. Ongoing research is being carried out to study several forms of dynamic connections, such as transesterification, imine, and disulfide linkages.^261^ Another current study is computational design and theoretical knowledge. Theoretical frameworks and computer simulations (*e.g.*, molecular dynamics and Monte Carlo simulations) are being applied to anticipate the behavior of vitrimers under diverse situations.^261,262^ This study highlights the chemistry of improving crosslinking and bond exchange rates, which are very crucial for tuning the properties of the targeted material. Vitrimers are also explored in several domains of biology, like tissue engineering, drug delivery, *etc.* Their adjustable mechanical qualities, biocompatibility, and capacity to self-heal make them attractive candidates for biodegradable implants and other medical devices. Recently, nanofillers have been introduced into vitrimer matrices to increase their mechanical, thermal, and electrical characteristics. Nanofillers are typically employed because they boost mechanical strength, thermal conductivity, self-healing characteristics, recyclability, and reprocessability. The success of nanofillers in vitrimers is primarily related to their high surface area-to-volume ratio, which allows for improved interaction with the polymer matrix. The use of nanofillers in vitrimer matrices provides various advantages. However, several problems must be addressed to ensure optimum compatibility, including inadequate dispersion, which leads to agglomeration and results in inconsistent material characteristics. Secondly, weak interfacial adhesion between nanofillers and the vitrimer matrix might limit load transmission and impair mechanical performance.^212^ Thirdly, the high viscosity of vitrimer matrices at processing temperatures can complicate the incorporation of nanofillers. Nanofillers can alter the thermal stability of vitrimers, potentially leading to degradation or changes in dynamic exchange reactions that are essential for their reprocessability.^263^

### 8.1 Strategies to overcome compatibility challenges

To enhance compatibility between nanofillers and vitrimers, several strategies can be employed, including surface modification of nanofibrils, such as chemical modification, which enhances the interaction within the matrices, and surface coating, which increases the dispersion and adhesion within the vitrimers. The second one optimizes the formulation of vitrimers by choosing the correct monomer, which promotes favorable interaction and modifies crosslink density during synthesis, which may influence how well nanofillers integrate into the network, enhancing mechanical performance. Further new processing methods may assist better integration, such as *in situ* polymerization, and high-energy mixing techniques can help achieve greater dispersion of nanofillers inside the vitrimer matrix, minimizing agglomeration. Nanofillers play a significant role in increasing the characteristics of vitrimer matrices, making them appropriate for many sophisticated applications. However, concerns relating to dispersion, interface adhesion, processing difficulties, and thermal stability must be addressed by strategic methods such as surface modification, formulation optimization, sophisticated processing techniques, and detailed characterization. By solving these hurdles, researchers may unleash the full potential of vitrimer-nanocomposite materials for future applications.

Vitrimers are a type of polymer that combine the qualities of thermoplastics and thermosets, allowing them to be molded and reprocessed several times without losing their mechanical capabilities. This capacity is due to dynamic covalent bonds that allow for bond exchange reactions, making vitrimers a possible option for managing plastic and thermoset composite wastes and encouraging a circular economy. As global polymeric waste increases day by day, scaling up vitrimer-based recycling processes is crucial for converting unwanted polymers into useable commodities. Thermal deterioration, phase separation, low adhesion, and limited compatibility with chemical recycling all make thermoplastic polyolefins (TPOs) difficult to recycle. Vitrimerization addresses these challenges by forming dynamic covalent connections, increasing recyclability, and improving durability without degradation. This provides vitrimerized polyolefins, a more sustainable and high-performance alternative to traditional TPOs for recycling and material design.^264,265^

The technique is meant to be easy and affordable, permitting the conversion of huge quantities of discarded polymers into reusable vitrimer networks while limiting resource consumption and expenses. Second is the utilization of waste feedstock. Several researchers have studied the synthesis of vitrimers utilizing waste materials such as polyurethane glycolysate. In the glycosylation of polyurethane or polymer wastes, the generated vitrimers have shown a commendable self-repairing capability. This strategy not only recycles existing resources but also promotes the sustainability of the process by minimizing dependency on virgin feedstocks.^266^ Further, to attain industrial scalability, recycling methods must enable high-throughput operations. Optimizing reaction conditions and equipment to allow the effective conversion of end to life polymers into vitrimers is critical. This entails creating technologies that can handle varied feedstocks and achieve consistent quality at scale.^145^ Nowadays, the use of 3D printing technology provides a novel approach for upcycling vitrimer materials. This technology enables the direct manufacture of components from recycled materials, decreasing waste and allowing tailored applications while harnessing the special features of vitrimers.^260^ So, scaling up vitrimer-based recycling has tremendous potential to eliminate polymeric waste while developing high-performance materials appropriate for different uses. By concentrating on robust chemical transformations, improving high-throughput processes, integrating sophisticated manufacturing methods, and overcoming current hurdles, the industrial application of vitrimers may contribute considerably to sustainable material solutions in the future. Continued research and cooperation among academics, industries, and the government will be crucial to fulfill this promise successfully.

### 8.2 Recyclability and reprocessability

Vitrimers can be degraded at the molecular level, which enables the effective recycling of composite materials without altering their mechanical properties and robustness. This special feature of vitrimers is crucial for reducing waste and confronting environmental concerns associated with plastics, which are commonly used for landfilling just after a single use.

### 8.3 Closed-loop material cycles

The ability of vitrimers to be completely recycled without significant loss of properties shows their potential for production in closed-loop cycles. Products manufactured from vitrimers may be developed, utilized, recycled, and rebuilt continually, hence lowering the demand for fresh raw materials and minimizing waste formation, which is the fundamental criterion for a circular economy.

### 8.4 Sustainable manufacturing practices

Vitrimers help eco-friendly production processes by enabling manufacture at lower temperatures and without the generation of volatile organic compounds (VOCs). This makes them a cleaner option than typical plastics and composites. The incorporation of biobased vitrimers further promotes sustainability by employing renewable resources, thereby lowering dependency on fossil fuels.^96^

### 8.5 Upcycling potential

Vitrimerization might help in the upcycling process of polymer composites into high-value products. Converting waste plastics into useful commodities greatly helps develop a sustainable circular economy. The adaptability of vitrimers enables them to be developed for many applications, from packaging to automobile components, therefore increasing their effect on sustainability initiatives.

## 9. Remarks

Vitrimerization is a game-changing invention in polymer nanocomposite recycling that provides practical, long-term solutions to some of the most critical waste management challenges. This technique is pioneering in the domain of sustainable materials science because it leverages dynamic covalent bonding to deliver low-energy recycling methods that retain material strength and functionality. One of the key benefits of vitrimerization is its compatibility with a wide spectrum of nanofillers, which increases desirable attributes such as self-healing, mechanical strength, and thermal stability. The study demonstrated that recycled vitrimer nanocomposites perform similarly or better than new materials, proving their potential for demanding, long-term applications. In line with international efforts to promote sustainability, vitrimerization has a substantially beneficial environmental impact in addition to its performance, lowering carbon emissions and saving resources. As the industry grows, overcoming concerns like higher manufacturing volumes and enhanced compatibility with diverse nanofillers will be vital. With the potential to revolutionize recycling and material design, vitrimerization is paving the way for circular economies in materials science, as well as a more sustainable and resource-efficient future.

Future studies must address a number of fascinating areas in order to fully realize the potential of vitrimer-based nanocomposites. For practical uses, increasing production levels without sacrificing cost-effectiveness will be essential. Next-generation composites with improved mechanical and functional performance may result from improving the dispersion and compatibility of nanofillers. Furthermore, vitrimerization, as a green technology, will be further enhanced by optimizing processing conditions to reduce energy input. Reliability spanning several life cycles requires an understanding of vitrimer systems' long-term durability, reusability, and degradation mechanisms. Furthermore, investigating more complex features like environmental responsiveness, self-adaptive behavior, and shape memory effects could put vitrimer nanocomposites at the forefront of high-performance and intelligent materials. By tackling these issues, polymer science and engineering will move more quickly towards a sustainable, circular future.

## Authors contributions

Sourav Ghosh: conceptualization, data curation, formal analysis, writing – original draft, and writing – review and editing; Amrita Chatterjee: writing – original draft and writing – review and editing; Nilanjan Dey: writing – original draft and writing – review and editing; Sunidhi Mishra: visualization, writing – original draft, and writing – review and editing; Shakshi Bhardwaj: writing – original draft and writing – review and editing; Shiva Singh: writing – original draft and writing – review and editing; Ujjal Tewary: visualization and writing – review and editing; Satyam Sahay: visualization and writing – review and editing; Madhuchhanda Maiti: supervision and validation; Pradip K. Maji: resources, software, supervision, validation, visualization, writing – original draft, and writing – review and editing.

## Conflicts of interest

The authors affirm that they have no known financial or interpersonal conflicts that would have appeared to impact the review presented in this study.

## Data Availability

No primary research results, software or code have been included and no new data were generated or analysed as part of this review.
